# Multiple model triangulation to identify factors associated with lameness in British sheep flocks

**DOI:** 10.1016/j.prevetmed.2021.105395

**Published:** 2021-08

**Authors:** K.E. Lewis, M.J. Green, J. Witt, L.E. Green

**Affiliations:** a School of Life Sciences, Gibbet Hill, Warwick University, Coventry, United Kingdom; b School of Veterinary Medicine and Science, University of Nottingham, Sutton Bonington Campus, Leicestershire, United Kingdom; c Institute of Microbiology and Infection, College of Life and Environmental Sciences, University of Birmingham, Birmingham, United Kingdom

**Keywords:** SFR, severe footrot, ID, interdigital dermatitis, Lameness, Sheep, Lambs, Footrot, Modelling, Statistical triangulation

## Abstract

Identification of factors associated with an outcome can be challenging when the number of explanatory variables is large in relation to the number of observations. Multiple model triangulation, where results from several model types are combined, improves the likelihood of identifying true predictor variables. The aim of this study was to use triangulation to identify covariates likely to be truly associated with the prevalence of lameness in sheep flocks in Great Britain.

Data were collected using a questionnaire sent to 3200 sheep farmers in Great Britain in 2018. The useable response rate was 14.1 %. The geometric mean prevalence of lameness was 1.4 % (95 % CI 1.2−1.7) for ewes, and 0.6 % (95 % CI 0.5−0.9) for lambs, however, approximately 60 % flocks had >2% prevalence of lameness in ewes.

Four model types were investigated, two generalised linear models (negative binomial and quasi-Poisson) built using stepwise selection, and two elastic net models (Poisson and Gaussian distributions) refined with selection stability estimation.

Triangulated covariates were those selected in three or all four models – 10 for ewes and 12 for lambs. Higher prevalence of lameness in ewes was associated with 5−100% feet bleeding during routine foot trimming compared with not foot trimming, footbathing the flock to treat severe footrot (SFR) and always using formalin in footbaths, both compared with not footbathing, using FootVax™ for <1 year compared with not using FootVax™, and never quarantining new or returning sheep to the farm for >3 weeks compared with always. Lower prevalence of lameness in ewes was associated with vaccinating with FootVax™ for >5 years compared with not vaccinating, peat soil compared with no peat soil, and having no lame ewes to treat.

Higher prevalence of lameness in lambs was associated with 5−100% feet bleeding during routine foot trimming, always foot trimming ewes with SFR, not knowingly selecting replacement ewes from ewes that were never lame compared with always, replacement sheep purchased and homebred compared with only homebred, treating lambs >3 days after recognition of lameness compared with 0-3 days and footbathing the flock to treat interdigital dermatitis compared with not footbathing at all. Lower prevalence of lameness in lambs was associated with peat soil, flocks in Scotland versus England, an altitude of >230−500 m compared with ≤230 m, never using antibiotic injection to treat lambs with SFR compared with always, and having no lame lambs to treat.

We conclude triangulation identified reliable management practices for farmers to implement to minimise lameness in sheep.

## Highlights

• Multiple model triangulation identifies variables that are likely true positives.
• Triangulation increases confidence in which managements to recommend in practice.
• Effective management of ewes can lower prevalence of lameness in ewes and lambs.

## 1 Introduction

Epidemiological research includes identification of factors associated with known health conditions, which can be challenging when analysing ‘wide’ data such as questionnaires when the number of explanatory variables is typically large in relation to the number of observations. Different model structures, analytic workflows, and variable selection techniques can give rise to different covariate selection because of the method used (Botvinik-Nezer et al., 2020; Lima et al., 2020a, 2021; Tercerio, 2003), raising questions for users on how to choose a modelling workflow and to improve the reproducibility of results. Triangulation of multiple methods is a recent concept that increases confidence in results (Lawlor et al., 2016; Lima et al., 2020a) by incorporating the uncertainty in each different methodology into variable selection and so providing more robust results (Lima et al., 2021).

Several studies have reported statistical associations between the prevalence of lameness in sheep flocks and management practices using retrospective postal and online questionnaires, typically requesting an estimate of the average proportion of lame sheep in the flock, flock size and management practices over a time period (Angell et al., 2014; Best et al., 2020; Dickins et al., 2016; Kaler and Green, 2009; Prosser et al., 2019; Reeves et al., 2019; Wassink et al., 2004; Winter et al., 2015). The majority of lameness in sheep is caused by footrot, an infectious bacterial disease present in >90.5 % of sheep flocks in England (Winter et al., 2015). Footrot initially presents as an interdigital dermatitis (ID) that can progress to severe footrot (SFR) when the hoof horn separates from the living dermis. There are many management practices associated with prevalence of lameness including recognition of lame sheep, intention to treat lame sheep, time to treatment of lame sheep, type of treatment, vaccination, footbathing, foot trimming and poor flock biosecurity (Best et al., 2020; Dickins et al., 2016; Kaler and Green, 2009; Prosser et al., 2019; Wassink et al., 2003, Wassink et al., 2004; Winter et al., 2015).

Variable selection is a key component for model creation. Traditional methods of variable selection are test-based (Desboulets, 2018), for example stepwise selection (Dohoo et al., 2003) in a generalised linear model (GLM) framework. Manual stepwise selection allows users to assess model combinations (Shtatland et al., 2008) but can result in inflated coefficient estimates, and false positive associations, particularly when models are over fitted or when there are multiple correlations between variables (Kuhn and Johnson, 2013; Hastie et al., 2015). Whilst methods have been proposed to control for correlated variables in GLM models such as selection of the most biologically relevant variable, use of a statistically determined cut-off value to remove highly correlated variables, or including both variables in a model where they are not collinear (Belsley et al., 1980), in practice, these decisions are arbitrary.

Overfitting is a particular challenge for ‘wide’ data and can arise when there are complex correlation structures as, for example, with management practices related to lameness in sheep - recognition of when a sheep is lame, whether a farmer catches a sheep for treatment and the type of treatment administered are in a complex correlation network (e.g. Winter et al., 2015) and so the risk of overfitting models is high. Overfitting leads to non-reproducible results in a wider population and therefore misleading information, in this example, on the importance of some management practices to control lameness.

There are a range of approaches to reduce overfitting and inflation of covariates. These include penalised regression models (Kuhn and Johnson, 2013) and bootstrap methods with regularised regression which allow calculation of additional metrics to ensure robust variable selection. One such metric is covariate stability (Austin and Tu, 2004; Hastie et al., 2015; Heinze et al., 2018; Meinshausen and Bühlmann, 2010), calculated as the proportion of times a covariate is selected by a model repeatedly run on subsamples of the data. Stability helps to discriminate true positive explanatory variables from “noise” variables (Austin and Tu, 2004; Lima et al., 2021).

Triangulation is a more recent concept to further enhance confidence in covariate selection (Lawlor et al., 2016; Lima et al., 2020a). The purpose of triangulation is to integrate results from several model types, each with different assumptions or sources of possible bias, to derive a more reliable answer (Lawlor et al., 2016). When variables are selected from different approaches, confidence in them is strengthened (Lima et al., 2020a). The concept of triangulation applies to the comparison of results between different modelling methods, since model outputs are influenced by both model choice (Tercerio, 2003; Ver Hoef and Boveng, 2007) and method of variable selection (Lima et al., 2020a).

The aim of the current study was to use triangulation of multiple model types, including those using adjustment for overfitting, to identify a robust set of covariates associated with the prevalence of lameness on sheep farms in Great Britain. Four model types were triangulated; two GLM models (negative binomial and quasi-Poisson) built with a stepwise selection process, and two elastic net models (Poisson and Gaussian distributions) refined with bootstrap stability estimation.

## 2 Materials and methods

### 2.1 Questionnaire design and administration

Ethical approval (reference number BSREC 67/18–19) was granted by the University of Warwick. The aim of the questionnaire (designed by JW and LG) was to collect updated figures for flock level prevalence of lameness in ewes and lambs, and their association with management practices and to widen the target population of sheep flocks from England only to include Welsh and Scottish flocks. The questionnaire (Supplementary File 2) had six sections – causes of lameness, patterns of lameness, management of the flock, culling and replacement of ewes and farm, and flock, characteristics. Questions were mostly closed, with some options for free text answers.

In 2018, 2000 paper questionnaires were sent by post to a random sample of farmers in England selected by the Agricultural and Horticultural Development Board (AHDB) that were registered on their database, and a further 600 farmers in Scotland, and 600 in Wales selected by the National Sheep Association (NSA). Two reminder letters were sent. There is no standard technique for sample size estimation for statistical methods based on bootstrap stability selection so we used conventional procedures to estimate sample size. For this, the prevalence of lameness in ewes and lambs was assumed to be 4.7 %, with -/+ 1 standard deviations corresponding to 1.4 and 7.8 %, and to follow a lognormal distribution. Based on a power of 0.8, significance probability of 0.05 and a set of possible effect sizes and variability in prevalence of lameness (Supplementary Table S15), it was estimated that a sample size of 500 flocks was needed. To allow for a relatively high non-response rate, the number of questionnaires sent to farmers was 3200.

### 2.2 Data cleaning and re-structuring of explanatory variables

Data were double entered by Wyman Dillon Ltd, Bristol, returned and stored as an Excel file, and cleaned manually by KL and JW, checking each response for errors and inconsistencies against the original questionnaire.

Questionnaires were useable when farmers reported the annual period prevalence of lameness in ewes and lambs, and the number of ewes and lambs in the flock (450 responses), and questions were useable if they were answered by >85 % of farmers. Where >85 % but not all farmers answered a question a “missing” category was created, for continuous variables the data were categorised into quintiles with a sixth “missing” category and for categorical variables one category was “missing” data. Use of this “missing” category resulted in dataset of 310 completely answered responses used for modelling work.

Data management and analyses were conducted using RStudio v3.6.0 (R statistical software, R Core Team, 2019). Descriptive statistics, measures of central tendency and dispersion and frequency distributions, were used to explore each variable and to inform recoding of variables for analysis. There were 57 categorical variables which were coded as 105 dummy variables for the elastic net models (Kassambara, 2018) using *fastDummies* (Kaplan, 2020). Associations between variables were explored using contingency tables and chi-square tests of association.

### 2.3 Models of associations between management practices and the prevalence of lameness in ewes

#### 2.3.1 Model types 1 and 2: generalised linear models

Two model structures appropriate for over-dispersed count data, the quasi-Poisson (QP-GLM) and negative binomial (NB-GLM), were used.

The models took the form:

$${\text{N}\text{u}\text{m}\text{b}\text{e}\text{r}\;\text{o}\text{f}\;\text{l}\text{a}\text{m}\text{e}\;\text{e}\text{w}\text{e}\text{s}}_{i}\;∼\;\text{α}\;+\text{o}\text{f}\text{f}\text{s}\text{e}\text{t}(\text{log}\left({\text{n}\text{u}\text{m}\text{b}\text{e}\text{r}\;\text{o}\text{f}\;\text{e}\text{w}\text{e}\text{s}\;\text{i}\text{n}\;\text{f}\text{l}\text{o}\text{c}\text{k}}_{i}\right))+\;\sum {\text{β}}_{\text{i}}{\text{X}}_{\text{i}}+\text{e}\;$$

where ∼ is the log link function, α the intercept, i the ith flock offset by the natural log of the number of ewes in the flock i and ${\text{β}}_{\text{i}}$ the coefficients for a series of predictor variables, ${\text{X}}_{\text{i}}$, and e the residual error. Confidence intervals were obtained by profiling the likelihood using *MASS* (Venables and Ripley, 2002).

Initially, four models were built using subsets of the variables (treatment of ewes and lambs, management of the flock, replacement of the flock, and the flock environment). Country and flock size were forced into each model. For the NB-GLM, a manual forwards stepwise selection process (Dohoo et al., 2009) was used to select variables for inclusion in the model using the *MASS* package (Venables and Ripley). For the quasi-Poisson model manual selection and the *stats* base package was used (R Core Team, 2019). Variables remained in the sub-models when the p-value from a Wald’s test of significance was <0.10.

Two final multivariable models were built from the sub-models using a forwards stepwise approach with variables retained in the model when p < 0.05 (Wald’s test). All variables were re-tested in the final multivariable model to check for residual confounding (Cox and Wermuth, 1996) and interactions between variables in the final model were checked, to be included if biologically relevant and significant (p < 0.05). Model fit was checked by ranking predicted and observed numbers of lame sheep per flock and summing them in deciles and comparing the distributions of the deciles (Cameron and Trivedi, 2013). Since model fit indicated that that the adjusted Poisson models did not correct sufficiently for over dispersion of the outcome variable, an additional dummy variable was created that identified flocks in the tenth decile as “problem flocks” – with a prevalence of lameness in ewes ≥7.1 % and in lambs ≥8.5 %. The “problem flock” variable was forced into the final models to evaluate model fit and retained where model fit was improved and it did not impact on the coefficients of other variables in the model.

#### 2.3.2 Model types 3 and 4: elastic net models with covariate selection stability

Because the specification of the response variable can influence model results (Tercerio, 2003) two distributions were used for model triangulation. These were:

1) A Poisson distribution with the outcome number of lame ewes in the flock, offset by the natural logarithm of the number of ewes in the flock (Poisson Elastic Net Bootstrap, “PEN-BS”)
2) A Gaussian log10(x+1) with the outcome log10((1+the number of lame ewes)/number of ewes in the flock), giving a rate (Gaussian Elastic Net Bootstrap, “GEN-BS”)

Models were fitted using the *glmnet* (Friedman et al., 2009) and *caret* R packages (Kuhn, 2020). The elastic net is designed to implement a balance between ridge regression and the least absolute shrinkage and selection operator (LASSO) penalties (Friedman et al., 2010). Full details of the model algorithms is in Friedman et al. (2010), but essentially the elastic net solves the problem:

$${SSE}_{net}=\;\frac{1}{2n}\sum_{i}^{n}{\left(y_{i}-\;{\text{ŷ}}_{i}\right)}^{2}+\;\;\lambda [\sum_{j}^{P}\left(\frac{1}{2}\left(1-\alpha \right)\beta_{j}^{2}\right)+\;\alpha \beta_{j}]$$

Where, for the Gaussian family, SSE_enet_ is the elastic net loss function to be minimised, i represents each farm, n the number of farms, $y_{i}$ the observed outcome for the ith farm and ${\text{ŷ}}_{i}$ the predicted outcome for the ith farm. The penalisation parameter is λ, with j, a predictor variable, p the total number of predictor variables, and α the mixing parameter that defined the relative proportion of penalisation on either the sum of the square of the coefficients (β^2^) or the unsquared coefficients (β).

For the Poisson regression model, *glmnet* uses an outer Newton loop, and an inner weighted least-squares loop to optimise the penalised log likelihood, using the equation:

$${\text{m}\text{i}\text{n}}_{\beta o,\;\beta }-\frac{1}{N}\text{l}(\text{β}\left|\text{X},\text{Y}\right)+\;\lambda \left(\left(1-\;\alpha \right)\;\sum_{i=1}^{N}{\text{β}}_{i}^{2}/2)+a\;\sum_{i=1}^{N}|{\text{β}}_{i}|\right)$$

Three further parameters were calculated for these models using a bootstrap procedure of 100 resamples (Hastie et al., 2015):

- Covariate stability: the percentage of times a covariate was selected in the elastic net model over the 100 bootstrap samples
- Coefficient 95 % confidence intervals (Steyerberg, 2019): the 2.5 and 97.5 percentile values from the distribution of covariate coefficients from the bootstrap samples when the variable was selected
- Bootstrap p-values: the smaller proportion of a coefficient’s values on one side of zero across the 100 bootstrap samples. For example, if a covariate was selected in the model in 80 of 100 bootstrap samples (i.e. a stability of 80 %) and 10 of these were all greater (or all less) than zero, then the bootstrap p-value would be 10/80 = 0.125.

For each elastic net model, from each of the 100 bootstrap samples, ten-fold cross validation, repeated 10 times was used to find the values λ and α (from a wide grid of parameter values) that minimised model mean absolute error (MAE). Values for α for both models ranged from 0.1 to 1.0 at 0.1 increments, and values for λ ranged from 0−30 for the PEN-BS model and 0–2 for the GEN-BS model, with distributions of the optimal value from each sample stored after each run to ensure a sufficient range had been used. The distribution of parameter values used are provided in Supplementary Material Fig. S1.

A cut-point selection stability of >80 % and a bootstrap p-value of <0.05 were chosen to identify predictor variables retained in the final model (Lima et al., 2020b).

A similar methodological approach was taken to identify predictor variables most consistently associated with prevalence of lameness in lambs. The four model types used were the same as those used to model the ewe data but using the number of lame lambs per flock as the numerator and number of lambs born as the denominator for the outcome variable.

## 3 Results

### 3.1 Response rate and flock characteristics of ewes and lambs in flocks in Great Britain, 2018

A total of 523 (16.3 %) questionnaires were returned, with 450 containing the average prevalence of lameness in ewes and lambs and the flock size, a useable response rate of 14.1 %. The useable response rate was reasonably similar by country – England – 15.2 %, Scotland 11.7 %, Wales – 12.7 %. There were 310 responses that were useable for modelling purposes (9.7 %). The geographical distribution of respondents is in Supplementary Fig. S2.

Flocks in Scotland were larger than flocks in England and Wales (Table 1) and some factors differed significantly between the three countries; including altitude, exposure to clay soil, an open flock and proportion of flocks vaccinating ewes with FootVax™ (MSD Animal Health) (Supplementary Table S2).

**Table 1:** Flock size and prevalence of lameness in ewes and lambs in 450 flocks of sheep in Great Britain (October 2017–September 2018).

	Overall	England	Scotland	Wales
**Flock characteristics (number)**				
Responses	450	304	70	76
Ewes (median, 95 % CI)	250 (220–300)	200 (165–235)^a^	545 (375–650)^b^	325 (230–500)^a^
Ewes (range)	4–5000	4–5000	4–2400	5–1800
Lambs born (median)	420 (350–490)	319 (270–400)^a^	775 (600–900)^b^	500 (350–700)^a^
Lambs born (range)	5–7500	6–7500	5–3546	10–2200
**Prevalence of lameness - ewes**				
Geometric mean % (95 % CI)	1.4 (1.2–1.7)	1.5 (1.2–1.9)	1.2 (0.8–1.9)	1.1 (0.6–1.7)
Median % (95 % CI)	2.0 (2.0–2.5)	2.4 (2.0–2.9)	2.0 (1.3–2.0)	2.0 (1.5–2.9)
Range %	0–39	0–39	0–30	0–15
**Prevalence of lameness - lambs**				
Geometric mean % (95 % CI)	0.6 (0.5–0.9)	0.6 (0.4–0.9)	0.5 (0.2–1.2)	1.0 (0.5–2.0)
Median % (95 % CI)	2.0 (2.0–2.1)	2.0 (2.0−2.7)	1.6 (1.0–2.0)	2.0 (1.9–3.0)
Range (%)	0–80	0–80	0–50	0–13

^abc^Superscripts indicate significant (Benjamini-Hochberg adjusted p-value ≤0.05) difference between countries, by post-hoc Wilcoxon tests.

*CI = confidence interval.

The geometric mean prevalence of lameness was 1.4 % (95 % CI 1.2−1.7) of ewes and 0.6 % (95 % CI 0.5−0.9) of lambs (Table 1), with a moderate within flock correlation between the prevalence of lameness in ewes and lambs (Spearman’s rank correlation rho = 0.60, p < 0.001). Infectious bacterial diseases were the predominant cause of lameness in both ewes and lambs, 87.8 % of farmers reported interdigital dermatitis, 75.3 % reported severe footrot and 36.9 % reported contagious ovine digital dermatitis (Supplementary Table S1).

### 3.2 Triangulation of associations between management practices and prevalence of lameness in ewes

The NB-GLM selected the fewest predictor variables (8), followed by the QP-GLM (13), the PEN-BS (17), with most selected by the GEN-BS (24), although the number selected by the latter two is determined by the threshold bootstrap value selected. The final model for each method is in Supplementary Tables S3–S6 and a visual assessment of fit of the generalised linear models in Supplementary Fig. S3.

**Fig. 1:**
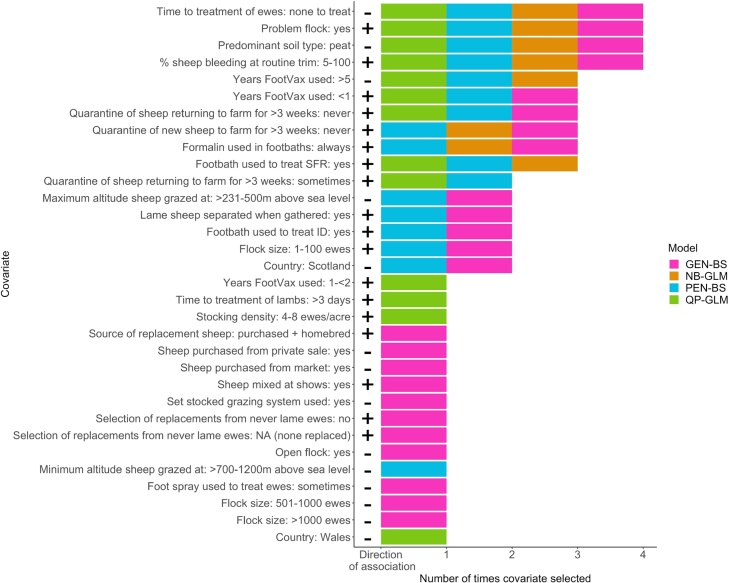
The number of times covariates were selected in final models for association with prevalence of lameness in ewes for the four model types ((Quasi-Poisson GLM (QP-GLM), Negative Binomial GLM (NB-GLM) boot-strapped Poisson models (PEN-BS) and Gaussian log(x+1) model (GEN-BS). Predictors that were not selected at all are not shown.

Triangulation across model types identified ten variables associated with the prevalence of lameness. Only four variables were selected in all four model types and six in three of four models of ewes (Fig. 1, Table 2). It was noticeable that the estimates and confidence intervals for each variable were similar across statistical methods (Table 2).

**Table 2:** Covariates associated with prevalence of lameness in ewes selected by triangulation in three or four of four model types (Quasi-Poisson generalised linear model, Negative binomial generalised linear model, bootstrap Poisson Elastic net and bootstrap Gaussian elastic net) in 310 flocks of sheep in Great Britain from October 2017–September 2018.

Covariate	N	%	QP-GLM	NB-GLM	PEN-BS	GEN-BS
			RR (95 % CI)	RR (95 % CI)	RR (95 % CI)	Coefficient (95 % CI)
**Problem Flock (Decile 10 -** ≥**7.14% lameness)**						
No	279	90.0	Ref	Ref		
Yes	31	10.0	3.12 (2.67–3.62)	3.72 (2.99–4.65)	2.89 (2.25–4.06)	0.42 (0.33–0.49)
**Predominant soil type - peat**						
No	265	85.5	Ref	Ref		
Yes	45	14.5	0.77 (0.65–0.90)	0.79 (0.65–0.95)	0.82 (0.66–0.98)	−0.08 (−0.16 to −0.00)
**Time to treatment of ewes with SFR**						
0−3 days	165	53.2	Ref	Ref		
>3 days	141	45.5				
None to treat	4	1.3	0.07 (0.00–0.41)	0.08 (0.01–0.29)	0.43 (0.15–0.83)	−0.49 (−0.86 to −0.27)
**% sheep feet bleeding at routine trim**						
Did not trim	115	37.1	Ref	Ref		
0	50	16.1				
>0–<5	104	33.5				
5–100	41	13.2	1.31 (1.13–1.51)	1.32 (1.07–1.62)	1.36 (1.17–1.60)	0.11 (0.04–0.19)
**Footbath to treat SFR**						
No	230	74.2	Ref	Ref		
Yes	80	25.8	1.27 (1.12–1.42)	1.17 (1.01–1.36)	1.13 (1.00–1.38)	
**Formalin used in footbaths**						
Did not footbath	66	21.3	Ref	Ref		
Always	85	27.4		1.36 (1.07–1.73)	1.12 (1.01–1.23)	0.04 (0.00–0.19)
Sometimes	79	25.5				
Never	80	25.8				
**Quarantine sheep returning to farm for >3 weeks**						
Always	60	19.4	Ref	Ref		
Sometimes	49	15.8				
Never	94	30.3	1.27 (1.07–1.50)		1.17 (1.03–1.38)	0.05 (0.01–0.12)
Missing	107	34.5				
**Quarantine new sheep to farm for >3 weeks**						
Always	162	52.3		Ref		
Sometimes	56	11.0				
Never	58	18.7		1.28 (1.06–1.55)	1.17 (1.02–1.42)	0.07 (0.00–0.14)
Did not purchase	34	18.1				
**Years FootVax™ used**						
Did not vaccinate	219	70.6	Ref	Ref		
<1	10	3.2	1.56 (1.27–1.89)		1.42 (1.09–1.84)	0.17 (0.07–0.37)
1–<2	19	6.1				
2–5	32	10.3				
>5	30	9.7	0.75 (0.60–0.92)	0.72 (0.57–0.90)	0.84 (0.69–0.99)	

N = number of flocks, RR = risk ratio, CI = confidence interval, QP-GLM = quasi-Poisson generalised linear model, NB-GLM = negative binomial generalised linear model, PEN-BS = Poisson elastic net model run on bootstrap data, GEN-BS = Gaussian elastic net model run on bootstrap data, SFR = severe footrot, ID = interdigital dermatitis, Ref = reference category.

The extra parameter to adjust for high prevalence of lameness was selected in all four models. In addition, in all four models there was a higher prevalence of lameness associated with 5−100% feet bleeding during routine foot trimming compared with not foot trimming at all. There was a lower prevalence of lameness when farmers reported no lame ewes to treat compared with treating lame ewes in 0−3 days. Flocks where sheep were kept on peat soil compared with no peat soil also had a lower prevalence of lameness.

Variables associated with a higher prevalence of lameness in three of the four models (Fig. 1, Table 2) were footbathing the flock to treat SFR and always using formalin in footbaths both compared with not footbathing at all, vaccination of sheep with FootVax™ for <1 year compared with not using Footvax™ at all, and never quarantining new or returning sheep for >3 weeks, compared with always doing so. A lower prevalence of lameness was associated with flocks vaccinated with FootVax™ for >5 years, compared with not using FootVax™ at all.

### 3.3 Triangulation of associations between management practices and prevalence of lameness in lambs

The QP-GLM selected the fewest predictor variables (16), followed by the NB-GLM (19), the PEN-BS (23) with most selected by the GEN-BS (25). The full model for each method is in Supplementary Table S7–S10, with visual assessment of fit of the generalised linear models in Supplementary Fig. S4.

**Fig. 2:**
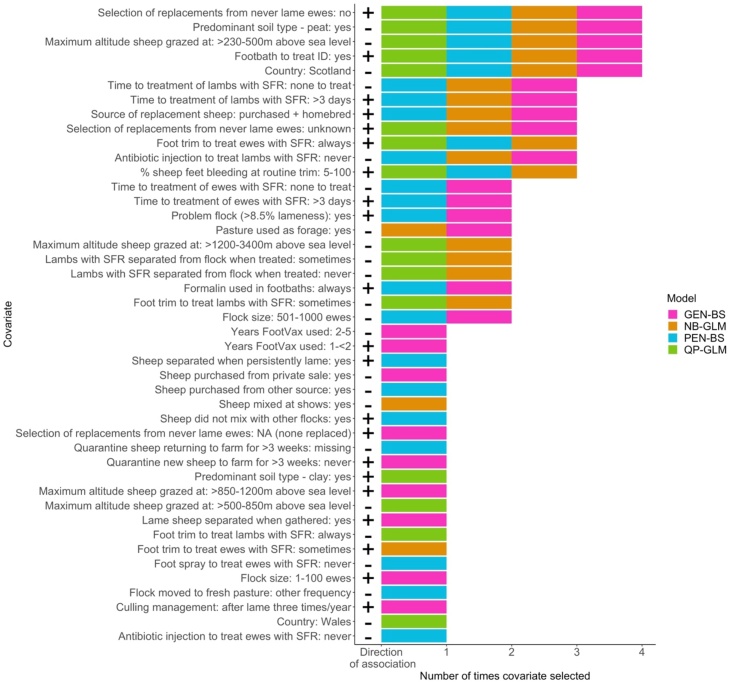
The number of times covariates were selected in final models for association with prevalence of lameness in lambs for the four model types ((Quasi-Poisson GLM (QP-GLM), Negative Binomial GLM (NB-GLM) boot-strapped Poisson models (PEN-BS) and Gaussian log(x+1) model (GEN-BS). Predictors that were not selected at all are not shown.

Triangulation identified 12 variables - five were selected in all four model types and a further seven in three of four models (Fig. 2, Table 3), fewer than in each model type. As for ewes, estimates and confidence intervals for each predictor variable were similar across statistical methods (Table 3).

**Table 3:** Covariates associated with prevalence of lameness in lambs selected by triangulation in three or four of four model types (Quasi-Poisson generalised linear model, Negative binomial generalised linear model, bootstrap Poisson Elastic net and bootstrap Gaussian elastic net) in 310 flocks of sheep in Great Britain from October 2017-September 2018.

Covariate	N	%	QP-GLM	NB-GLM	PEN-BS	GEN-BS
			RR (95 % CI)	RR (95 % CI)	RR (95 % CI)	Coefficient (95 % CI)
**Country**						
England	219	70.6	Ref	Ref		
Scotland	43	13.9	0.52 (0.35–0.75)	0.71 (0.52–0.96)	0.84 (0.66–0.97)	−0.07 (−0.19–−0.01)
Wales	48	15.5				
**Footbath to treat ID**						
No	170	54.8	Ref	Ref		
Yes	140	45.2	1.64 (1.25–2.17)	1.35 (1.09–1.68)	1.22 (1.07–1.57)	0.09 (0.03–0.18)
**Maximum altitude flock was grazed at (m above sea level)**						
0–230	52	16.8	Ref	Ref		
>230–500	52	16.8	0.49 (0.31–0.78)	0.69 (0.48–0.99)	0.86 (0.59–0.98)	−0.07 (−0.21–−0.00)
>500–850	61	19.7				
>850–1200	56	18.1				
>1200–3400	42	13.5				
Missing	47	15.2				
**Selection of replacements from ewes that were never lame**						
Yes	86	27.7	Ref	Ref		
No	87	28.1	2.07 (1.47–2.92)	1.77 (1.34–2.34)	1.25 (1.06–1.60)	0.08 (0.01–0.22)
Unknown	99	31.9	1.61 (1.15–2.27)	1.38 (1.04–1.84)		0.05 (0.00–0.15)
Not applicable	38	12.3				
**Predominant soil type - peat**						
No	265	85.5	Ref	Ref		
Yes	45	14.5	0.53 (0.35–0.78)	0.64 (0.48–0.87)	0.84 (0.68–0.98)	−0.08 (−0.19–−0.01)
**% sheep feet bleeding at routine foot trim**						
Did not foot trim	115	37.1	Ref	Ref		
0	50	16.1				
>0–<5	104	33.5				
5–100	41	13.2	1.91 (1.34–2.72)	1.48 (1.07–2.07)	1.19 (1.01–1.48)	
**Foot trim to treat ewes with SFR**						
Never	51	16.5	Ref	Ref		
Sometimes	97	31.3				
Always	162	52.3	2.13 (1.24–3.68)	1.95 (1.26–3.01)	1.12 (0.98–1.25)	
**Antibiotic injection to treat lambs with SFR**						
Always	109	35.2		Ref		
Sometimes	136	43.9				
Never	65	21.0		0.71 (0.53–0.95)	0.92 (0.71–1.00)	−0.05 (−0.15–0.00)
**Source of replacement sheep**						
Homebred	164	52.9		Ref		
Purchased	42	13.5				
Homebred + purchased	94	30.3		1.55 (1.21–1.97)	1.12 (0.98–1.26)	0.07 (0.01–0.13)
Not applicable	10	3.2				
**Time to treatment of lambs with SFR**						
0–3 days	161	51.9		Ref		
>3 days	131	42.3		1.51 (1.22–1.87)	1.15 (1.02–1.35)	0.06 (0.01–0.15)
None to treat	18	5.8		0.04 (0.01–0.12)	0.66 (0.12–0.95)	−0.37 (−0.61–−0.15)

N = number of flocks, RR = risk ratio, CI = confidence interval, QP-GLM = quasi-Poisson generalised linear model, NB-GLM = negative binomial generalised linear model, PEN-BS = Poisson elastic net model run on bootstrap data, GEN-BS = Gaussian elastic net model run on bootstrap data, SFR = severe footrot, ID = interdigital dermatitis, Ref = reference category.

Three of the variables associated with lower prevalence of lameness in lambs were environmental - flocks kept on peat soil compared with no peat, flocks in Scotland compared with England and flocks grazed at >230−500 m above sea level compared with ≤230 m. Two of the variables associated with a lower prevalence of lameness in lambs were managemental - never using antibiotic injection to treat lambs with SFR compared with always, and having no lame lambs to treat compared with treating lame lambs in 0−3 days. However, treating lambs >3 days after recognition of lameness compared to within 0−3 days was associated with a higher prevalence of lameness.

Ewe management practices associated with a higher prevalence of lameness in lambs were: 5−100% of ewes bleeding during routine foot trimming compared with not foot trimming at all; always foot trimming ewes with SFR compared with never doing so; not knowingly selecting replacement ewes from ewes that were never lame compared to always doing so; and replacement sheep both purchased and homebred compared with only homebred. One flock variable was associated with a higher prevalence of lameness in lambs, this was footbathing the flock to treat ID compared with not footbathing at all.

### 3.4 Variable stability

**Fig. 3:**
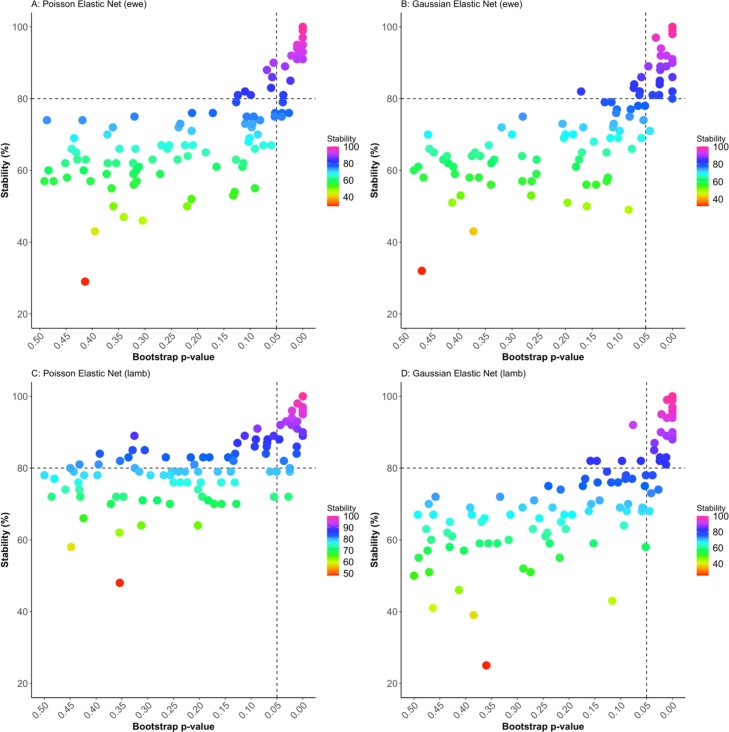
Stability (the proportion of times the predictor was selected by the elastic net model in the 100 boot-strapped samples) vs boot-strap p-value (the proportion of times the coefficient for the predictor was > or < than 0 (depending on the median coefficient) in the Poisson (A) and Gaussian (B) elastic net models for management practices associated with prevalence of lameness in ewes, and in Poisson (C) and Gaussian (D) elastic net models for management practices associated with prevalence of lameness in lambs in 310 sheep flocks in Great Britain from October 2017-September 2018.

In the elastic net bootstrapped models for both ewes and lambs, predictor variables with high stability tended to have lower p-values (Fig. 3) and so there was a clear demarcation of between variables that comprised the ‘final model’ and other variables both on stability and bootstrap p value.

## 4 Discussion

Our study is the first to implement multiple model triangulation to identify robust associations between farm management practices and the prevalence of lameness in sheep flocks. Previous triangulation of models in animal health used continuous outcome data (Lima et al., 2020b), our results indicate that triangulation is equally useful with Poisson models: three of the four models were for count data (Ver Hoef and Boveng, 2007), whilst one assumed a loglinear function. Triangulation highlighted a small set of variables selected in three or four model types (Figs. 1 and 2). These variables are therefore likely to be the most reliable management practices associated with prevalence of lameness in this sample and more likely to be informative for the population of sheep flocks in Great Britain because triangulation reduces the impact of bias from each modelling method, strengthening confidence that selected covariates have a true association with the outcome and would be reproduceable (Lawlor et al., 2016).

Some of the triangulated variables in our study have been reported in previous studies whilst others are new. In addition, analysing ewe and lamb data in separate models has highlighted that some management practices for ewes, and the whole flock, influence the prevalence of lameness in lambs. We can also learn from management of footrot by disease severity, because lambs are less likely to develop SFR than ewes (Supplementary Table S1) and so risks for lambs with ID might equate to risks for ewes with SFR. These are discussed below.

There was an increased risk of lameness in both ewes and lambs when 5−100% of sheep feet bled after routine foot trimming, and when foot trimming was part of treatment of ewes with SFR (Tables 2 and 3, Figs. 1 and 2). Feet bleeding during routine foot trimming has been associated with higher prevalence of lameness in ewes (Winter et al., 2015; Prosser et al., 2019) and foot trimming ewes with SFR delays healing (Kaler et al., 2010), consequently, it is consistent that these practices were associated with higher prevalence of lameness in ewes. However, it is less clear why foot trimming ewes was associated with a higher prevalence of lameness in lambs. Foot trimming lambs as a direct risk for increased prevalence of lameness was reported in Lewis and Green (2020) and in the current study farmers who foot trimmed ewes to treat SFR were more likely to also foot trim lambs as part of treatment for footrot (p < 0.01, Supplementary Table S13), and so it is possible that only the ewe variable was selected in the models. Alternatively, the risk to lambs might be indirect, because foot trimming may increase the prevalence of ewes with footrot (Kaler et al., 2010), which would increase spread of disease and so the incidence of footrot in ewes and lambs.

Another ewe variable, this time associated with lower prevalence of lameness in lambs was conscious selection of replacement ewes from dams that were never lame (Table 3). Such selection increases resistance or resilience to footrot which is mildly heritable (Nieuwhof et al., 2008; Raadsma et al., 1994; Skerman et al., 1988). The results indicate that closed flocks could derive benefits in control of lameness from such planned selection programmes.

There was one environmental factor associated with lameness in both ewes and lambs. The prevalence of lameness was lower in ewes and lambs in flocks on predominately peat compared with no peat soil (Tables 2 and 3). Peat has a lower pH than other soil types (Wheeler et al., 2010), which could affect survival of *D. nodosus* or other bacteria in the foot and so change the interdigital skin microbial community. A laboratory study (Muzafar et al., 2015) reported longer survival of *D. nodosus* in clay rich soils, indicating some difference in survival by soil type, but peat soils were not included in that study. However, there are other plausible explanations for this association. For example, flocks on peat are also likely to be at low stocking density because it is marginal land, and low stocking density is associated with lower prevalence of footrot (Wassink et al., 2003, Kaler and Green, 2009). Flock management might also explain the lower prevalence of lameness in lambs in flocks in Scotland compared with England and Wales as Scottish flocks were larger and on higher ground (Table 1, Supplementary Table S2). One other environmental factor was associated with a lower prevalence of lameness in lambs. This was when flocks were kept at a maximum altitude of >230m-500 m above sea level compared with ≤230 m. A similar association between altitude and prevalence of ID was reported by (Wassink et al., 2004). However, as with peat soils, higher altitudes are associated with marginal land, lower air temperature, and low stocking density, which are all associated with lower prevalence of footrot (Wassink et al., 2003, Wassink et al., 2004)

Analysing data for lambs and ewes separately increased insight into good management practices to control lameness in lambs and ewes. In the current study, footbathing to treat ID and SFR was associated with a higher prevalence of lameness in lambs and ewes respectively, compared with not using footbaths at all. Lambs rarely develop SFR (Supplementary Table S1) and so ID is the common presenting sign of footrot, whereas ewes do develop SFR from ID. These results highlight that treating any stage of footrot with footbaths is less effective than individual rapid treatment of lame sheep, or indeed not having any lame sheep to treat. This is probably both because farmers delay treatment until sufficient sheep are lame to use a footbath (Kaler and Green, 2009) but also because footbaths are not an effective treatment of SFR (Wassink et al., 2010a). Overall, our paper has highlighted that footbathing is not an effective management to minimise footrot in lambs or ewes.

Our study provides the first evidence that formalin footbaths are associated with a higher prevalence of lameness in ewes than other footbath products. Footbathing with formalin has been associated with flock-presence of shelly hoof and foot granulomas (Reeves et al., 2019). Granulomas are very painful and affected ewes are lame (Winter et al., 2015), given that the geometric mean prevalence of granuloma lesions in ewes in affected flocks in the current study was 0.8 % and the mean prevalence of lameness overall was 1.4 %, these lesions could account for much of higher prevalence of lameness in those flocks using formalin. Of the 152 farmers that reported sheep with granulomas, 86.2 % used a footbath, with 31.6 % always using formalin, while of the 147 who reported no granulomas, 71.4 % used a footbath, and only 23.1 % always used formalin, this was significantly fewer farmers using formalin (Fisher’s exact test, p = 0.02, Supplementary Table S11).

The complex risk pattern associated with time since starting to vaccinate with FootVax™ (a lower risk when >5 years, an increased risk when <1 year and no difference when vaccination had been used 2−5 years) was identified via triangulation in ewe models but not lamb models. This association with ewes was first reported in 2019 (Prosser et al., 2019) and then by Best et al. (2020). Only 20.9 % of farmers vaccinated ewes, with only 2.9 % for <1 year and 8.9 % for >5 years but the variable was robust in our triangulated approach, suggesting a real effect. Vaccinating ewes was not, however, associated with a lower prevalence of lameness in lambs. This suggests that lambs were not protected from footrot indirectly by vaccinated ewes.

Never quarantining new or returning sheep for >3 weeks, compared with always doing so were associated with a higher prevalence of lameness in ewes, as in Winter et al. (2015); 20.0 % of farmers always quarantined returning stock for > 3 weeks. Footrot is highly endemic (Prosser et al., 2020) but the robustness of this risk indicates that there is still a benefit from quarantine for > 3 weeks. This might be because quarantine prevents the introduction of new strains of *D. nodosus* to a flock and also reduces the risk of introducing contagious ovine digital dermatitis, another infectious casue of lameness (Dickins et al., 2016).

There were a small number of flocks with no lame ewes (4) or lambs (18). Not surprisingly, but very encouragingly, these flocks had a lower period prevalence of lameness than flocks with lame ewes, even if treated within 3 days of becoming lame. Despite this, our study highlights, for the first time, that treatment of lame lambs within 3 days of onset of lameness was associated with a lower prevalence of lameness than treatment after 3 days. This has been reported previously in ewes, where rapid treatment is the highest attributable risk to maintain a low prevalence of lameness (Grant et al., 2018; Prosser et al., 2019). Our study supports this management practice in lambs.

Whilst treating lambs >3 days after recognising lameness compared with 0−3 days was selected by the triangulation process in lamb models, the equivalent practice in ewes was not in the ewe models (Fig. 1), suggesting that time to treatment was not as reliable a variable in the ewe models as in the lamb models. One explanation for this is that time to treatment of lambs was more consistent than for ewes. This might be because lambs remain on farm for 4–6 months and are handled regularly and so regular treatment is given, whilst ewes management varies throughout the production year and e.g. some farmers do not treat lame ewes during pregnancy (O’Kane et al., 2017) or when harvesting (Witt and Green, 2018). This might indicate that that question needs refining for future studies to allow for variable answers across the production cycle.

Flocks where lambs with SFR were never treated with antibiotic injection to treat lame lambs had a lower prevalence of lameness than flocks where lambs were always treated with antibiotic injection – this was also reported in Lewis and Green (2020). Current Sheep Veterinary Society guidelines only recommend treating lambs with antibiotic injection if clinical signs of SFR are present, and to use antibiotic foot spray alone for signs of ID (Sheep Veterinary Society, 2013). Our questionnaire did not ask about recognition of lameness – recognition of lameness only at high locomotion scores was identified as a risk factor for higher prevalence of lameness in lambs (Lewis and Green, 2020) and it is possible that the farmers who never used antibiotic injection were treating lambs promptly with foot spray, recognising lame lambs at low locomotion scores and that this was sufficient to prevent progression to SFR and the need to use antibiotic injection in lambs.

The geometric mean prevalence of all lameness was low in the current study conducted in 2018, ewes - 1.4 % (95 % CI 1.2−1.7) and lambs - 0.6 % (95 % CI 0.5−0.9) compared with previous estimates in English sheep flocks of 4.1 % in 2015 (Prosser et al., 2019), and 3.5 % in 2013 (Winter et al., 2015). The summer of 2018 was unusually dry (Met Office, 2018) which would have reduced the prevalence of footrot (Clifton et al., 2019; Graham and Egerton, 1968; Smith et al., 2014). It would be interesting to see estimates from a wet summer to see how well footrot is controlled in conditions conducive to spread of disease. However, if the flocks in the current study are representative of flocks in Great Britain, then the FAWC (Farm Animal Welfare Council, 2011) target of a national flock prevalence of lameness of <2% by 2021 is getting closer to being achieved. However, even in 2018 approximately 60 % of flocks had >2% lameness (Supplementary Table S14b).

Standard limitations of questionnaire studies apply to this research. One limitation of cross-sectional studies is determining causality. Sheep farmers rarely change their management practices (Wassink et al., 2010a) and therefore management practices in 2018 are likely to be those used in 2017, strengthening the likelihood that associations between management practices and prevalence of lameness are temporally likely to be causal. In addition, other study types have identified similar associations (Kaler et al., 2010; Wassink et al., 2010b; Witt and Green, 2018).

The response proportion was lower than other paper-based studies (Kaler and Green, 2009; Winter et al., 2015). This is an increasing trend in the livestock industry and might be because of the number of questionnaires and forms farmers are now asked to complete.

Statistical triangulation is robust to selection of false positive covariates (Lima et al., 2020a), however, there is the possibility of omitting true variables i.e. false negative covariates. Our results indicate that our original sample size estimates (Supplementary Material Table S15) were conservative because smaller effect sizes than expected were detected (Table 2). These effect sizes were quite small e.g. risk ratios ≥1.12 with an exposure proportion of 27.4 % of flocks, so although our response number was 310 rather than 500 questionnaires, we conclude that 310 was sufficient to identify management practices that are clinically and economically important risks for lameness. Covariates with smaller effect sizes than 1.12, or that occurred on a small proportion of farms might have been missed, however, removing these risks would contribute little to reduction of lameness in the national flock.

In conclusion, our study illustrates that triangulation of results from different model types identifies a robust set of variables associated with prevalence of lameness in ewes and lambs. Some of these associations have been associated with prevalence of lameness previously, while others are reported for the first time. These risks are likely to be the most reliable for reduction of prevalence of lameness on sheep farms since multiple model triangulation reduces the likelihood of false positive associations.

## Funding

Kate Lewis is funded by a Biotechnology and Biological Sciences Research Council Midlands Integrative Biosciences Training Partnership iCase studentship with the Agriculture and Horticulture Development Board and Jessica Witt is funded by a Biotechnology and Biological Sciences Research Council Midlands Integrative Biosciences Training Partnership studentship.

## Data statement

Data will not be made publicly available as consent from respondents to do so was not obtained at the time of collection.

## Declaration of Competing Interest

The authors report no declarations of interest.
