# Supplementary material for: Multiple model triangulation to identify factors associated with lameness in British sheep flocks
Source: Prev Vet Med. 2021 Aug;193:105395. doi: 10.1016/j.prevetmed.2021.105395 (PMC8326248; doi:10.1016/j.prevetmed.2021.105395)
Supplement: Supplementary file 2 [file mmc2.pdf]

Farm ID: ******

Questionnaire: Lameness management and disease prevalence in English sheep flocks

Section 1. Causes of lameness

Please use the descriptions and pictures below to identify causes of lameness and answer the related questions.

Cause and lesion description	Example picture of the lesion	1. Did this lesion cause lameness in your flock between October 2017 - September 2018 ?<br>*Please circle one answer and fill in %s for each box below*
**Scald/early footrot**<br>• Red, wet interdigital space<br>• Foul smelling, grey, pasty scum<br>• Loss of hair in interdigital space	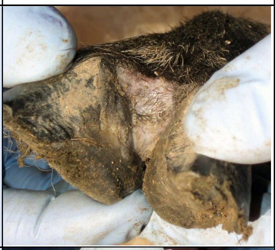	**Yes** **No** **Do not know**<br>***If yes,* what percent were lame from this lesion on any given day?**<br>Ewes __________% Lambs __________%
**Footrot**<br>• Some separation of horn from underlying foot<br>• Foul smelling, slimy, dead tissue	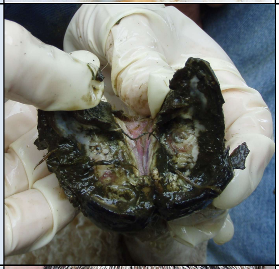	**Yes** **No** **Do not know**<br>***If yes,* what percent were lame from this lesion on any given day?**<br>Ewes __________% Lambs __________%
**CODD**<br>• Loss of hair at coronary band<br>• Red lesion prone to bleeding<br>• May have complete detachment of hoof	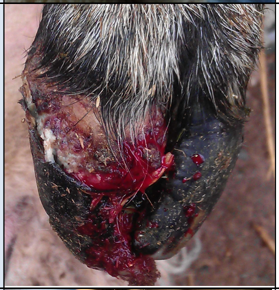	**Yes** **No** **Do not know**<br>***If yes,* what percent were lame from this lesion on any given day?**<br>Ewes __________% Lambs __________%
**Granuloma**<br>• Strawberry-like growth, frequently at the toe<br>• Sometimes hidden under overgrown horn<br>• Bleeds when handled	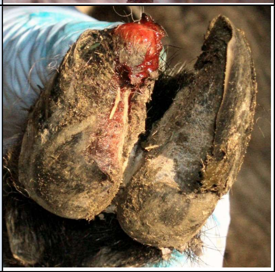	**Yes** **No** **Do not know**<br>***If yes,* what percent were lame from this lesion on any given day?**<br>Ewes __________% Lambs __________%
**Shelly hoof**<br>• Pockets of separation of hoof from foot<br>• No foul smell or dead tissue<br>• Can become impacted with soil and stones	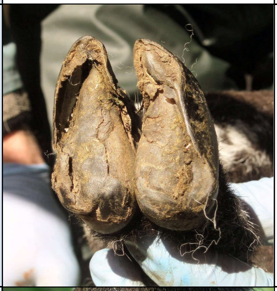	**Yes** **No** **Do not know**<br>***If yes,* what percent were lame from this lesion on any given day?**<br>Ewes __________% Lambs __________%
**White line abscess**<br>• Foot may be hot and painful with no outward signs of disease<br>• May burst at the coronary band releasing pus	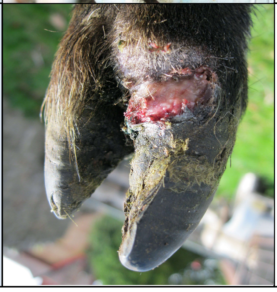	**Yes** **No** **Do not know**<br>***If yes,* what percent were lame from this lesion on any given day?**<br>Ewes __________% Lambs __________%

Section 2. Patterns of lameness

2. Please complete the table below with the percentage of ewes lame with footrot each month

Oct 2017	Nov 2017	Dec 2017	Jan 2018	Feb 2018	Mar 2018	Apr 2018	May 2018	June 2018	July 2018	Aug 2018	Sept 2018

3. Please complete the table below with the percentage of ewes lame with scald each month

Oct 2017	Nov 2017	Dec 2017	Jan 2018	Feb 2018	Mar 2018	Apr 2018	May 2018	June 2018	July 2018	Aug 2018	Sept 2018

4. Please complete the table below with the percentage of lambs lame with footrot each month

Oct 2017	Nov 2017	Dec 2017	Jan 2018	Feb 2018	Mar 2018	Apr 2018	May 2018	June 2018	July 2018	Aug 2018	Sept 2018

5. Please complete the table below with the percentage of lambs lame with scald each month

Oct 2017	Nov 2017	Dec 2017	Jan 2018	Feb 2018	Mar 2018	Apr 2018	May 2018	June 2018	July 2018	Aug 2018	Sept 2018

6. In your experience, which of the environmental factors listed below contribute to the spread of footrot and scald in your flock? *(Please circle all that apply)*

Footrot: **Daily rainfall** **Daily temperature** **Day length** **Wind speed**

Soil type **Soil moisture** **Altitude** **Other** *(Please specify)* ____________________________

Scald: **Daily rainfall** **Daily temperature** **Day length** **Wind speed**

Soil type **Soil moisture** **Altitude** **Other** *(Please specify)* ____________________________

7. Between October 2017 – September 2018, which specific factors not listed above contributed to the spread of footrot and scald in your flock?

Section 3. Management of lameness

8. Between October 2017 - September 2018, when you saw lame <u>ewes</u> how soon did you usually treat them? *(Please circle <u>one</u>)*

The first day you saw them	Within three days	Within one week	Within two weeks	Longer than two weeks	Did not treat any lame sheep

9. Between October 2017 - September 2018, when you saw lame <u>lambs</u> how soon did you usually treat them? *(Please circle <u>one</u>)*

The first day you saw them	Within three days	Within one week	Within two weeks	Longer than two weeks	Did not treat any lame sheep

10. Between October 2017 - September 2018, which of the following did you <u>always</u> use to treat footrot in ewes and lambs? *(Please circle <u>all that apply</u>)*

**Ewes:**	**Antibiotic injection**	**Foot spray**	**Foot trimming**	**Separate lame individuals**	**Other treatment** *(Please specify)* ____________________
**Lambs:**	**Antibiotic injection**	**Foot spray**	**Foot trimming**	**Separate lame individuals**	**Other treatment** *(Please specify)* ____________________

11. Between October 2017 - September 2018, which of the following did you <u>never</u> use to treat footrot in ewes and lambs? *(Please circle <u>all that apply</u>)*

**Ewes:**	**Antibiotic injection**	**Foot spray**	**Foot trimming**	**Separate lame individuals**	**Other treatment** *(Please specify)* ____________________
**Lambs:**	**Antibiotic injection**	**Foot spray**	**Foot trimming**	**Separate lame individuals**	**Other treatment** *(Please specify)* ____________________

12. Between October 2017 and September 2018, which of the following did you do in your flock as part of management of lameness? *(Please circle <u>all that apply</u>)*

Routine foot trimming	Routine foot bathing	Culling lame sheep	Separation of lame sheep	Select replacements from never lame ewes	Other *(Please specify)* ____________

13. Approximately what percentage of sheep bled when you did a routine trim? ______% **Not applicable**

14. Did you use formalin in footbaths? **Always** **Sometimes** **Never** **Not applicable**

15. Why did you use a footbath? **Routine practice when gathered** **To treat footrot** **To treat scald** **Not applicable**

16. When did you separate lame sheep? *(Please circle <u>all that apply</u>)*

Never	When an individual sheep was treated	When the group was gathered (weaning, e.g.)	When persistently lame	Other *(Please specify)* ____________________

17. Which of your sheep did you vaccinate with Footvax between October 2017 - September 2018? (*Please circle <u>all that apply</u>*)

None	Ewes	Rams	Sheep with footrot	Replacements	Other *(Please specify)* ____________________

If None, please go to <u>Section 4</u>

18. How often did you vaccinate with Footvax? *(Please circle one)*

Once a year **Every 6 months** **Before periods when high footrot levels were expected**

19. When did you start vaccinating against footrot? *(Please circle one)*

Within the last year **Within the last 2 years** **Within the last 5 years** **More than 5 years ago**

Section 4. Culling and replacements

20. Between October 2017 – September 2018, when did you cull sheep that had been lame? *(Please circle one)*

Never **After lame twice in the year** **After lame 3 times in the year** **After persistently lame** **Other** *(Please specify)* ______________________________

21. What was the source for your replacements? *(Please circle one)*

Home bred **Purchased** **Both** **Not applicable**

If Purchased only, please go to Question 20

22. Did you select replacements from ewes that were never lame? *(Please circle one)*

Yes **No** **Don't know** **Not applicable**

23. Which type of sheep did you purchase between October 2017 - September 2018? *(Please circle all that apply)*

None **Rams** **Ewes** **Store lambs** **Other** *(Please specify)* ______________________

24. Where did you purchase your sheep from? *(Please circle all that apply)*

Dealer/agent **Market** **Private farm sale** **Other** *(Please specify)* ____________________

25. Between October 2017 - September 2018, did you inspect new sheep arriving on your farm for lameness? *(Please circle one)*

Always **Sometimes** **Never**

26. Between October 2017 - September 2018, did you quarantine new sheep arriving on your farm for at least 3 weeks? *(Please circle one)*

Always **Sometimes** **Never**

27. Between October 2017 - September 2018, did you quarantine sheep returning to your farm (from shows, loaned rams, etc.) for at least 3 weeks? *(Please circle one)*

Always **Sometimes** **Never**

Section 5. Grazing and the environment

28. Between October 2017 – September 2018, did your flock mix with other flocks? *(Please circle all that apply)*

No **Yes, shared grazing** **Yes, at shows** **Yes, through broken fences** **Other** *(Please specify)* ________________________

29. On average, what stocking rate did you use between October 2017 - September 2018? (*Please circle one*)

Less than 4 ewes per acre **4-8 Ewes per acre** **More than 8 ewes per acre**

30. How many acres did you use for grazing your flock between October 2017 - September 2018? __________acres

31. What types of forage did the flock graze on? *(Please circle all that apply)*

Pasture **Forage crops** **Aftermath** **Other** *(Please specify)* _________________

32. Over the last 4 years, were pastures resown? *(Please circle one)*

All pastures resown **Some pastures resown and some permanent pasture** **All are permanent pastures**

33. What grazing method did you use most frequently? *(Please circle all that apply)*

Rotational grazing **Set stocked** **Other** *(please specify)* ___________________________

34. On average, how frequently was the flock moved to fresh pasture? *(Please circle one)*

More than once a week **Once a week** **Once every 2 weeks** **Other** *(Please specify)* __________________

35. What are the predominant soil types in your grazing pastures? *(Please circle all that apply)*

Clay **Loamy** **Sandy** **Peaty**

36. What was the **minimum** altitude that you grazed your flock? ________________________ feet above sea level

37. What was the **maximum** altitude that you grazed your flock? ________________________ feet above sea level

Section 6. Flock details

38. What is the postcode* for the farm where the flock is kept? ______________________

**This information will be used ONLY for modelling disease patterns, not for correspondence*

39. Between October 2017 - September 2018 …

   a. How many ewes did you have in your breeding flock? ________
   b. How many lambs were -
      - Born ________
      - Weaned ________
      - Sold ________
      - Kept for breeding ________
   c. What was the average level of lameness in ewes in your flock? ________%
   d. What was the average level of lameness in lambs in your flock? ________%
   e. What was the highest level of lameness in ewes in your flock? ________%
   f. What was the highest level of lameness in lambs in your flock? ________%

40. In the previous year (October 2016 – September 2017) …

   a. What was the average level of lameness in ewes in your flock? ________%
   b. What was the average level of lameness in lambs in your flock? ________%

Comments: If there are any other lameness issues that you wish to address, please discuss them in the box below.

Section 7: Contact information and further research

41. If you would like to receive the results from this survey and others, please provide us with an email address

Email: ____

Would you like to receive a summary of the results of this questionnaire?	**Yes**	**No**
Would you like to receive a summary of the results of our other research?	**Yes**	**No**

42. If you would like to participate in further research into lameness or mastitis in sheep, please fill in your contact details below:

Name: ___________________________________ Postcode: ________________________________

Address: _________________________________

_________________________________ Telephone: _______________________________

____

____

____

Are you interested in helping with our future research into lameness in sheep? (*Please circle all that apply)*

Yes, questionnaires **Yes, clinical trials** **No**

Are you interested in helping with our future research into mastitis in sheep? (*Please circle all that apply)*

Yes, questionnaires **Yes, clinical trials** **No**

PLEASE RETURN YOUR COMPLETED QUESTIONNAIRE IN THE FREEPOST ENVELOPE PROVIDED
