# Supplementary material for: Multiple model triangulation to identify factors associated with lameness in British sheep flocks
Source: Prev Vet Med. 2021 Aug;193:105395. doi: 10.1016/j.prevetmed.2021.105395 (PMC8326248; doi:10.1016/j.prevetmed.2021.105395)
Supplement: Supplementary file 1 [file mmc1.docx]

Supplementary Figure 1: the distribution of values of alpha and lambda used for hyperparameter tuning in the elastic net models for A) Poisson models and B) Gaussian models

Supplementary Figure 2: Location of the 227 flocks who supplied a valid postcode, coloured by the source of the questionnaire (AHDB – pink, NSA sent to Scotland – orange, NSA sent to Wales – blue).

Supplementary Table 1: The number of flocks affected by each type of foot lesion (interdigital dermatitis, severe footrot, contagious ovine digital dermatitis, granuloma, shelly hoof and white line abscess), the overall geometric mean prevalence of each foot lesion in Great Britain (October 2017-September 2018), and the geometric mean prevalence in flocks only where the foot lesion was reported.

| **Foot lesion** | **Farms affected (%)** | **Overall GM prevalence (%)** | **N** | **GM prevalence in farms where foot lesion present (%)** | **N** |
| --- | --- | --- | --- | --- | --- |
| *Interdigital dermatitis* | 87.8 |  |  |  |  |
| Ewes |  | 0.41 (0.26-0.63) | 408 | 2.53 (2.27-2.82) | 348 |
| Lambs |  | 0.50 (0.32-0.79) | 418 | 3.66 (3.28-4.08) | 353 |
| *Severe footrot* | 75.3 |  |  |  |  |
| Ewes |  | 0.09 (0.06-0.16) | 430 | 1.90 (1.69-2.13) | 324 |
| Lambs |  | 0.01 (0.00-0.01) | 389 | 2.32 (2.04-2.64) | 200 |
| *Contagious ovine digital dermatitis* | 36.9 |  |  |  |  |
| Ewes |  | 0.00 (0.00-0.00) | 426 | 1.64 (1.38-1.96) | 159 |
| Lambs |  | 0.00 (0.00-0.00) | 408 | 2.36 (1.84-3.03) | 90 |
| *Granuloma* | 46.9 |  |  |  |  |
| Ewes |  | 0.00 (0.00-0.00) | 428 | 0.81 (0.70-0.94) | 200 |
| Lambs |  | 0.00 (0.00-0.00) | 381 | 0.84 (0.47-1.49) | 20 |
| *Shelly hoof* | 58.7 |  |  |  |  |
| Ewes |  | 0.02 (0.01-0.03) | 421 | 2.04 (1.76-2.36) | 253 |
| Lambs |  | 0.00 (0.00-0.00) | 351 | 1.51 (1.18-1.93) | 79 |
| *White line abscess* | 30.7 |  |  |  |  |
| Ewes |  | 0.00 (0.00-0.00) | 401 | 1.10 (0.94-1.29) | 127 |
| Lambs |  | 0.00 (0.00-0.00) | 376 | 1.21 (0.89-1.64) | 46 |

1. N = number of flocks, GM = geometric mean, % = percentage of flocks using the denominator of 450 useable responses
2. Overall geometric mean prevalence calculated as including flocks where prevalence of the foot lesion was given as 0, adding 0.00001 to allow log transformation. Geometric mean prevalence in farms where foot lesion present was calculated as prevalence where the prevalence of the foot lesion was >0

Supplementary Table 2: The number and percentage of flocks in Great Britain where a management practice was performed between October 2017-September 2018

| **Predictor** | **Overall** | | **England** | | **Scotland** | | **Wales** | |
| --- | --- | --- | --- | --- | --- | --- | --- | --- |
|  | **N** | **%** | **N** | **%** | **N** | **%** | **N** | **%** |
| **Country** |  |  |  |  |  |  |  |  |
| England | 304 | 67.6 | 304 | 100.0 | - | - | - | - |
| Scotland | 70 | 15.6 | - | - | 70 | 100.0 | - | - |
| Wales | 76 | 16.9 | - | - | - | - | 76 | 100.0 |
| **Flock size (number of ewes)*** | | |  |  |  |  |  |  |
| 101-500 | 174 | 38.7 | 126 | 41.4 | 20 | 28.6 | 28 | 36.8 |
| 1-100 | 135 | 30.0 | 102 | 33.6 | 13 | 18.6 | 20 | 26.3 |
| 501-1000 | 96 | 21.3 | 54 | 17.8 | 21 | 30.0 | 21 | 27.6 |
| >1000 | 45 | 10.0 | 22 | 7.2 | 16 | 22.9 | 7 | 9.2 |
| **Time to treatment of ewes with SFR** | | | |  |  |  |  |  |
| 0-3 days | 232 | 51.6 | 155 | 51.0 | 34 | 48.6 | 43 | 56.6 |
| >3 days | 201 | 44.7 | 138 | 45.4 | 32 | 45.7 | 31 | 40.8 |
| None to treat | 9 | 2.0 | 6 | 2.0 | 3 | 4.3 | 0 | 0 |
| **Time to treatment of lambs with SFR** | | | |  |  |  |  |  |
| 0-3 days | 230 | 51.1 | 159 | 52.3 | 31 | 44.3 | 40 | 52.6 |
| >3 days | 184 | 40.9 | 118 | 38.8 | 33 | 47.1 | 33 | 43.4 |
| None to treat | 25 | 5.6 | 20 | 6.6 | 5 | 7.1 | 0 | 0 |
| **Antibiotic injection to treat ewes with SFR** | | | | | |  |  |  |
| Always | 229 | 50.9 | 151 | 49.7 | 36 | 51.4 | 42 | 55.3 |
| Never | 63 | 14.0 | 45 | 14.8 | 8 | 11.4 | 10 | 13.2 |
| Sometimes | 149 | 33.1 | 108 | 35.5 | 22 | 31.4 | 19 | 25 |
| **Foot trim to treat ewes with SFR** | | | | |  |  |  |  |
| Always | 222 | 49.3 | 157 | 51.6 | 32 | 45.7 | 33 | 43.4 |
| Sometimes | 154 | 34.2 | 104 | 34.2 | 26 | 37.1 | 24 | 31.6 |
| Never | 65 | 14.4 | 43 | 14.1 | 8 | 11.4 | 14 | 18.4 |
| **Foot spray to treat ewes with SFR** | | | | |  |  |  |  |
| Always | 360 | 80.0 | 250 | 82.2 | 54 | 77.1 | 56 | 73.7 |
| Sometimes | 68 | 15.1 | 44 | 14.5 | 10 | 14.3 | 14 | 18.4 |
| Never | 13 | 2.9 | 10 | 3.3 | 2 | 2.9 | 1 | 1.3 |
| **Ewes with SFR separated from flock when treated** | | | | | |  |  |  |
| Always | 81 | 18.0 | 53 | 17.4 | 17 | 24.3 | 11 | 14.5 |
| Sometimes | 154 | 34.2 | 104 | 34.2 | 21 | 30 | 29 | 38.2 |
| Never | 206 | 45.8 | 147 | 48.4 | 28 | 40 | 31 | 40.8 |
| **Footbath to treat SFR** | | | |  |  |  |  |  |
| No | 327 | 72.7 | 225 | 74.0 | 48 | 68.6 | 54 | 71.1 |
| Yes | 121 | 26.9 | 79 | 26.0 | 21 | 30 | 21 | 27.6 |
| **Footbath to treat ID** | | | | |  |  |  |  |
| No | 251 | 55.8 | 179 | 58.9 | 31 | 44.3 | 41 | 53.9 |
| Yes | 197 | 43.8 | 125 | 41.1 | 38 | 54.3 | 34 | 44.7 |
| **Antibiotic injection to treat lambs with SFR** | | | | |  |  |  |  |
| Always | 142 | 31.6 | 93 | 30.6 | 24 | 34.3 | 25 | 32.9 |
| Sometimes | 206 | 45.8 | 140 | 46.1 | 34 | 48.6 | 32 | 42.1 |
| Never | 93 | 20.7 | 71 | 23.4 | 8 | 11.4 | 14 | 18.4 |
| **Foot trim to treat lambs with SFR** | | | | |  |  |  |  |
| Always | 147 | 32.7 | 106 | 34.9 | 18 | 25.7 | 23 | 30.3 |
| Sometimes | 215 | 47.8 | 145 | 47.7 | 35 | 50.0 | 35 | 46.1 |
| Never | 79 | 17.6 | 53 | 17.4 | 13 | 18.6 | 13 | 17.1 |
| **Foot spray to treat lambs with SFR** | | | | |  |  |  |  |
| Always | 333 | 74.0 | 231 | 76 | 50 | 71.4 | 52 | 68.4 |
| Sometimes | 94 | 20.9 | 62 | 20.4 | 14 | 20.0 | 18 | 23.7 |
| Never | 14 | 3.1 | 11 | 3.6 | 2 | 2.9 | 1 | 1.3 |
| **Lambs with SFR separated from flock at treatment** | | | | |  |  |  |  |
| Always | 42 | 9.3 | 32 | 10.5 | 6 | 8.6 | 4 | 5.3 |
| Never | 199 | 44.2 | 141 | 46.4 | 28 | 40 | 30 | 39.5 |
| Sometimes | 200 | 44.4 | 131 | 43.1 | 32 | 45.7 | 37 | 48.7 |
| **Formalin used in footbaths** | | | | | |  |  |  |
| Did not footbath | 94 | 20.9 | 69 | 22.7 | 8 | 11.4 | 17 | 22.4 |
| Always | 122 | 27.1 | 83 | 27.3 | 25 | 35.7 | 14 | 18.4 |
| Sometimes | 115 | 25.6 | 75 | 24.7 | 18 | 25.7 | 22 | 28.9 |
| Never | 115 | 25.6 | 74 | 24.3 | 19 | 27.1 | 22 | 28.9 |
| **% sheep feet bleeding at routine foot trim** | | | | |  |  |  |  |
| Did not foot trim | 174 | 38.6 | 110 | 36.2 | 27 | 38.6 | 37 | 48.7 |
| 0 | 66 | 14.7 | 40 | 13.2 | 11 | 15.7 | 15 | 19.7 |
| >0-<5 | 138 | 30.6 | 105 | 34.5 | 20 | 28.6 | 13 | 17.1 |
| 5-100 | 61 | 13.6 | 40 | 13.2 | 9 | 12.9 | 10 | 13.2 |
| **Footbath used as routine practice** | | | |  |  |  |  |  |
| No | 300 | 66.7 | 208 | 68.4 | 45 | 64.3 | 47 | 61.8 |
| Yes | 148 | 32.9 | 96 | 31.6 | 24 | 34.3 | 28 | 36.8 |
| **Lame sheep never separated from flock** | | | |  |  |  |  |  |
| No | 279 | 62.0 | 191 | 62.8 | 44 | 62.9 | 44 | 57.9 |
| Yes | 169 | 37.6 | 113 | 37.2 | 25 | 35.7 | 31 | 40.8 |
| **Lame sheep separated from flock at treatment** | | | | |  |  |  |  |
| No | 386 | 85.8 | 266 | 87.5 | 58 | 82.9 | 62 | 81.6 |
| Yes | 62 | 13.8 | 38 | 12.5 | 11 | 15.7 | 13 | 17.1 |
| **Lame sheep separated from flock when gathered*** | | | | |  |  |  |  |
| No | 391 | 86.9 | 269 | 88.5 | 51 | 72.9 | 71 | 93.4 |
| Yes | 57 | 12.7 | 35 | 11.5 | 18 | 25.7 | 4 | 5.3 |
| **Sheep separated from flock when persistently lame** | | | | |  |  |  |  |
| No | 280 | 62.2 | 188 | 61.8 | 42 | 60 | 50 | 65.8 |
| Yes | 168 | 37.3 | 116 | 38.2 | 27 | 38.6 | 25 | 32.9 |
| **Flock did not mix with other flocks** | | | |  |  |  |  |  |
| No | 96 | 21.3 | 59 | 19.4 | 14 | 20 | 23 | 30.3 |
| Yes | 354 | 78.7 | 245 | 80.6 | 56 | 80 | 53 | 69.7 |
| **Flock mixed through others via shared grazing** | | | | |  |  |  |  |
| No | 413 | 91.8 | 286 | 94.1 | 65 | 92.9 | 62 | 81.6 |
| Yes | 37 | 8.2 | 18 | 5.9 | 5 | 7.1 | 14 | 18.4 |
| **Flock mixed with others at shows** | | | | | |  |  |  |
| No | 419 | 93.1 | 289 | 95.1 | 63 | 90 | 67 | 88.2 |
| Yes | 31 | 6.9 | 15 | 4.9 | 7 | 10 | 9 | 11.8 |
| **Flock mixed with others through broken fences** | | | | |  |  |  |  |
| No | 415 | 92.2 | 279 | 91.8 | 65 | 92.9 | 71 | 93.4 |
| Yes | 35 | 7.8 | 25 | 8.2 | 5 | 7.1 | 5 | 6.6 |
| **Stocking density** | | |  |  |  |  |  |  |
| <4 ewes/acre | 237 | 52.7 | 164 | 53.9 | 37 | 52.9 | 36 | 47.4 |
| 4-8 ewes/acre | 187 | 41.6 | 122 | 40.1 | 27 | 38.6 | 38 | 50 |
| >8 ewes/acre | 20 | 4.4 | 12 | 3.9 | 6 | 8.6 | 2 | 2.6 |
| **Pasture used as forage** | | |  |  |  |  |  |  |
| No | 5 | 1.1 | 4 | 1.3 | 1 | 1.4 | 0 | 0 |
| Yes | 445 | 98.9 | 300 | 98.7 | 69 | 98.6 | 76 | 100.0 |
| **Crops used as forage** | |  |  |  |  |  |  |  |
| No | 350 | 77.8 | 242 | 79.6 | 53 | 75.7 | 55 | 72.4 |
| Yes | 100 | 22.2 | 62 | 20.4 | 17 | 24.3 | 21 | 27.6 |
| **Aftermath used as forage*** | |  |  |  |  |  |  |  |
| No | 232 | 51.6 | 169 | 55.6 | 35 | 50 | 28 | 36.8 |
| Yes | 218 | 48.4 | 135 | 44.4 | 35 | 50 | 48 | 63.2 |
| **Resowing of pastures*** | |  |  |  |  |  |  |  |
| Mixed frequency | 236 | 52.4 | 135 | 44.4 | 48 | 68.6 | 53 | 69.7 |
| All permanent | 202 | 44.9 | 161 | 53 | 19 | 27.1 | 22 | 28.9 |
| All resown | 4 | 0.9 | 3 | 1 | 1 | 1.4 | 0 | 0 |
| **Use of rotational grazing system** | | | |  |  |  |  |  |
| No | 194 | 43.1 | 129 | 42.4 | 36 | 51.4 | 29 | 38.2 |
| Yes | 256 | 56.9 | 175 | 57.6 | 34 | 48.6 | 47 | 61.8 |
| **Use of set stocked grazing system** | | |  |  |  |  |  |  |
| No | 239 | 53.1 | 170 | 55.9 | 27 | 38.6 | 42 | 55.3 |
| Yes | 211 | 46.9 | 134 | 44.1 | 43 | 61.4 | 34 | 44.7 |
| **Frequency flock moved to fresh pasture*** | | | | | |  |  |  |
| Every two weeks | 147 | 32.7 | 100.0 | 32.9 | 20 | 28.6 | 27 | 35.5 |
| > once per week | 18 | 4.0 | 9 | 3 | 3 | 4.3 | 6 | 7.9 |
| Once per week | 43 | 9.6 | 20 | 6.6 | 6 | 8.6 | 17 | 22.4 |
| Other | 211 | 46.9 | 154 | 50.7 | 34 | 48.6 | 23 | 30.3 |
| **Predominant soil type – clay*** | | | |  |  |  |  |  |
| No | 217 | 48.2 | 127 | 41.8 | 47 | 67.1 | 43 | 56.6 |
| Yes | 233 | 51.8 | 177 | 58.2 | 23 | 32.9 | 33 | 43.4 |
| **Predominant soil type - loamy** | | | |  |  |  |  |  |
| No | 252 | 56 | 182 | 59.9 | 35 | 50 | 35 | 46.1 |
| Yes | 198 | 44 | 122 | 40.1 | 35 | 50 | 41 | 53.9 |
| **Predominant soil type - sandy** | | | |  |  |  |  |  |
| No | 368 | 81.8 | 247 | 81.2 | 59 | 84.3 | 62 | 81.6 |
| Yes | 82 | 18.2 | 57 | 18.8 | 11 | 15.7 | 14 | 18.4 |
| **Predominant soil type - peat** | | |  |  |  |  |  |  |
| No | 374 | 83.1 | 261 | 85.9 | 52 | 74.3 | 61 | 80.3 |
| Yes | 76 | 16.9 | 43 | 14.1 | 18 | 25.7 | 15 | 19.7 |
| **Sheep vaccinated with FootVax™** | | |  |  |  |  |  |  |
| No | 319 | 70.9 | 223 | 73.4 | 49 | 70 | 47 | 61.8 |
| Yes | 128 | 28.4 | 81 | 26.6 | 21 | 30 | 26 | 34.2 |
| **Ewes vaccinated with FootVax™*** | | | |  |  |  |  |  |
| No | 353 | 78.4 | 246 | 80.9 | 54 | 77.1 | 53 | 69.7 |
| Yes | 94 | 20.9 | 58 | 19.1 | 16 | 22.9 | 20 | 26.3 |
| **Rams vaccinated with FootVax™** | | |  |  |  |  |  |  |
| No | 356 | 79.1 | 247 | 81.2 | 54 | 77.1 | 55 | 72.4 |
| Yes | 91 | 20.2 | 57 | 18.8 | 16 | 22.9 | 18 | 23.7 |
| **Sheep with footrot vaccinated with FootVax™** | | | | | |  |  |  |
| No | 438 | 97.3 | 296 | 97.4 | 70 | 100.0 | 72 | 94.7 |
| Yes | 9 | 2.0 | 8 | 2.6 | 0 | 0 | 1 | 1.3 |
| **Replacements vaccinated with FootVax™** | | | |  |  |  |  |  |
| No | 380 | 84.4 | 259 | 85.2 | 60 | 85.7 | 61 | 80.3 |
| Yes | 67 | 14.9 | 45 | 14.8 | 10 | 14.3 | 12 | 15.8 |
| **Frequency sheep vaccinated with FootVax™** | | | | |  |  |  |  |
| Did not vaccinate | 314 | 69.8 | 218 | 71.7 | 49 | 70 | 47 | 61.8 |
| Once a year | 106 | 23.6 | 67 | 22 | 17 | 24.3 | 22 | 28.9 |
| Twice a year | 8 | 1.8 | 6 | 2 | 1 | 1.4 | 1 | 1.3 |
| Before footrot expected | 7 | 1.6 | 5 | 1.6 | 1 | 1.4 | 1 | 1.3 |
| **Years FootVax™ used** | | | |  |  |  |  |  |
| Did not vaccinate | 312 | 69.3 | 216 | 71.1 | 49 | 70.0 | 47 | 61.8 |
| <1 | 13 | 2.9 | 8 | 2.6 | 3 | 4.3 | 2 | 2.6 |
| 1-<2 | 24 | 5.3 | 17 | 5.6 | 0 | 0 | 7 | 9.2 |
| 2-5 | 45 | 10.0 | 33 | 10.9 | 7 | 10.0 | 5 | 6.6 |
| >5 | 40 | 8.9 | 23 | 7.6 | 7 | 10.0 | 10 | 13.2 |
| **Culling of lame sheep** | |  |  |  |  |  |  |  |
| Never | 121 | 26.9 | 80 | 26.3 | 18 | 25.7 | 23 | 30.3 |
| After lame twice per year | 42 | 9.3 | 26 | 8.6 | 13 | 18.6 | 3 | 3.9 |
| After lame three times per year | 37 | 8.2 | 24 | 7.9 | 6 | 8.6 | 7 | 9.2 |
| After persistently lame | 219 | 48.7 | 152 | 50 | 30 | 42.9 | 37 | 48.7 |
| Other | 14 | 3.1 | 8 | 2.6 | 2 | 2.9 | 4 | 5.3 |
| **Source of replacement sheep** | | | |  |  |  |  |  |
| Homebred | 231 | 51.3 | 144 | 47.4 | 41 | 58.6 | 46 | 60.5 |
| Purchased | 75 | 16.7 | 62 | 20.4 | 4 | 5.7 | 9 | 11.8 |
| Homebred + purchased | 119 | 26.4 | 79 | 26 | 23 | 32.9 | 17 | 22.4 |
| Not applicable | 16 | 3.6 | 11 | 3.6 | 1 | 1.4 | 4 | 5.3 |
| **Selection of replacements from ewes that were never lame** | | | | | |  |  |  |
| Yes | 111 | 24.7 | 76 | 25 | 21 | 30.0 | 14 | 18.4 |
| No | 122 | 27.1 | 75 | 24.7 | 27 | 38.6 | 20 | 26.3 |
| Unknown | 126 | 28.0 | 86 | 28.3 | 15 | 21.4 | 25 | 32.9 |
| Not applicable | 54 | 12.0 | 40 | 13.2 | 4 | 5.7 | 10 | 13.2 |
| **Open flock^+^** |  |  |  |  |  |  |  |  |
| No | 59 | 13.1 | 44 | 14.5 | 3 | 4.3 | 12 | 15.8 |
| Yes | 391 | 86.9 | 260 | 85.5 | 67 | 95.7 | 64 | 84.2 |
| **Sheep purchased from dealer** | | | |  |  |  |  |  |
| No | 432 | 96.0 | 291 | 95.7 | 68 | 97.1 | 73 | 96.1 |
| Yes | 15 | 3.3 | 13 | 4.3 | 1 | 1.4 | 1 | 1.3 |
| **Sheep purchased from market** | | |  |  |  |  |  |  |
| No | 175 | 38.9 | 130 | 42.8 | 21 | 30.0 | 24 | 31.6 |
| Yes | 272 | 60.4 | 174 | 57.2 | 48 | 68.6 | 50 | 65.8 |
| **Sheep purchased from private sale** | | | | |  |  |  |  |
| No | 290 | 64.4 | 195 | 64.1 | 43 | 61.4 | 52 | 68.4 |
| Yes | 157 | 34.9 | 109 | 35.9 | 26 | 37.1 | 22 | 28.9 |
| **Sheep purchased from other source** | | | |  |  |  |  |  |
| No | 426 | 94.7 | 292 | 96.1 | 65 | 92.9 | 69 | 90.8 |
| Yes | 21 | 4.7 | 12 | 3.9 | 4 | 5.7 | 5 | 6.6 |
| **New sheep inspected for lameness** | | | | |  |  |  |  |
| Always | 303 | 67.3 | 199 | 65.5 | 53 | 75.7 | 51 | 67.1 |
| Did not purchase | 44 | 9.8 | 31 | 10.2 | 3 | 4.3 | 10 | 13.2 |
| Sometimes | 47 | 10.4 | 34 | 11.2 | 8 | 11.4 | 5 | 6.6 |
| Never | 29 | 6.4 | 20 | 6.6 | 4 | 5.7 | 5 | 6.6 |
| **Quarantine new sheep for >3 weeks** | | | | | |  |  |  |
| Always | 232 | 51.6 | 151 | 49.7 | 45 | 64.3 | 36 | 47.4 |
| Did not purchase | 45 | 10.0 | 32 | 10.5 | 3 | 4.3 | 10 | 13.2 |
| Sometimes | 79 | 17.6 | 50 | 16.4 | 14 | 20.0 | 15 | 19.7 |
| Never | 73 | 16.2 | 53 | 17.4 | 8 | 11.4 | 12 | 15.8 |
| **Quarantine sheep returning to farm for >3 weeks*** | | | | |  |  |  |  |
| Always | 90 | 20.0 | 49 | 16.1 | 26 | 37.1 | 15 | 19.7 |
| Sometimes | 61 | 13.6 | 37 | 12.2 | 8 | 11.4 | 16 | 21.1 |
| Never | 126 | 28.0 | 96 | 31.6 | 16 | 22.9 | 14 | 18.4 |
| Missing | 173 | 38.4 | 122 | 40.1 | 20 | 28.6 | 31 | 40.8 |
| **Minimum altitude flock was grazed at (m above sea level)*** | | | | | |  |  |  |
| -5-100 | 88 | 19.6 | 57 | 18.8 | 20 | 28.6 | 11 | 14.5 |
| >100-286 | 57 | 12.7 | 38 | 12.5 | 8 | 11.4 | 11 | 14.5 |
| >286-450 | 78 | 17.3 | 52 | 17.1 | 14 | 20.0 | 12 | 15.8 |
| >450-700 | 92 | 20.4 | 52 | 17.1 | 14 | 20.0 | 26 | 34.2 |
| >700-1200 | 61 | 13.6 | 38 | 12.5 | 11 | 15.7 | 12 | 15.8 |
| Missing | 74 | 16.4 | 67 | 22 | 3 | 4.3 | 4 | 5.3 |
| **Maximum altitude flock was grazed at (m above sea level)*** | | | | | |  |  |  |
| 0-230 | 76 | 16.9 | 61 | 20.1 | 9 | 12.9 | 6 | 7.9 |
| >230-500 | 74 | 16.4 | 50 | 16.4 | 15 | 21.4 | 9 | 11.8 |
| >500-850 | 79 | 17.6 | 50 | 16.4 | 13 | 18.6 | 16 | 21.1 |
| >850-1200 | 85 | 18.9 | 45 | 14.8 | 17 | 24.3 | 23 | 30.3 |
| >1200-3400 | 63 | 14.0 | 32 | 10.5 | 13 | 18.6 | 18 | 23.7 |
| Missing | 73 | 16.2 | 66 | 21.7 | 3 | 4.3 | 4 | 5.3 |

^+^	Open flock defined as purchase of any sheep (ewes, replacement, store lambs or rams

* indicates where the number of flocks where the management practice was performed

differs between countries (p <0.05 from either chi-square test of association or Fisher’s

exact test with p-values simulated by Monte Carlo simulation, as appropriate).

1. All percentages were calculated using the number of useable responses (450) as the

denominator, therefore where the percentages do not sum to 100.0 across the predictor, some responses were missing. (Missing) indicates the “missing” category used where the question was answered by <85% of respondents.

2. SFR = severe footrot, ID = interdigital dermatitis

Supplementary Table 3: Multivariable Quasi-Poisson generalised linear model for the management practices associated with the proportion of lame ewes in 310 sheep flocks in Great Britain, October 2017-September 2018.

| **Predictor** | **N** | **%** | **RR** | **LCI** | **UCI** | **P-value** | |
| --- | --- | --- | --- | --- | --- | --- | --- |
| (Intercept) |  |  | 0.02 | 0.02 | 0.03 | <0.01 | |
| **Country** |  |  |  |  |  |  | |
| England | 219 | 70.6 | 1.00 |  |  |  | |
| Scotland | 43 | 13.9 | 0.85 | 0.72 | 1.00 | 0.05 | |
| **Wales** | **48** | **15.5** | **0.76** | **0.64** | **0.90** | **<0.01** | |
| **Flock size (number of ewes)** | | | |  |  |  | |
| 101-500 | 118 | 38.1 | 1.00 |  |  |  | |
| 1-100 | 91 | 29.4 | 1.26 | 0.98 | 1.60 | 0.07 | |
| 501-1000 | 72 | 23.2 | 0.90 | 0.78 | 1.04 | 0.15 | |
| >1000 | 29 | 9.4 | 0.91 | 0.77 | 1.08 | 0.26 | |
| **Problem flock (Decile 10 -** ≥**7.14% lameness)** | | | |  |  |  | |
| No | 279 | 90.0 | 1.00 |  |  |  | |
| **Yes** | **31** | **10.0** | **3.12** | **2.67** | **3.62** | **<0.01** | |
| **% sheep feet bleeding at routine foot trim** | | | |  |  |  | |
| Did not foot trim | 115 | 37.1 | 1.00 |  |  |  | |
| 0 | 50 | 16.1 | 0.82 | 0.63 | 1.05 | 0.12 | |
| >0-<5 | 104 | 33.5 | 0.90 | 0.78 | 1.03 | 0.12 | |
| **5-100** | **41** | **13.2** | **1.31** | **1.13** | **1.51** | **<0.01** | |
| **Years FootVax™ used** | |  |  |  |  |  | |
| Did not vaccinate | 219 | 70.6 | 1.00 |  |  |  | |
| **<1** | **10** | **3.2** | **1.56** | **1.27** | **1.89** | **<0.01** | |
| **1-<2** | **19** | **6.1** | **1.31** | **1.07** | **1.61** | **0.01** | |
| 2-5 | 32 | 10.3 | 0.90 | 0.77 | 1.06 | 0.22 | |
| **>5** | **30** | **9.7** | **0.75** | **0.60** | **0.92** | **<0.01** | |
| **Predominant soil type - peat** | | |  |  |  |  | |
| No | 265 | 85.5 | 1.00 |  |  |  | |
| **Yes** | **45** | **14.5** | **0.77** | **0.65** | **0.90** | **<0.01** | |
| **Footbath to treat SFR** | | |  |  |  |  | |
| No | 230 | 74.2 | 1.00 |  |  |  | |
| **Yes** | **80** | **25.8** | **1.27** | **1.12** | **1.42** | **<0.01** | |
| **Time to treatment of ewes with SFR** | | | | |  |  | |
| 0-3 days | 165 | 53.2 | 1.00 |  |  |  | |
| >3 days | 141 | 45.5 | 0.86 | 0.73 | 1.01 | 0.06 | |
| **None to treat** | **4** | **1.3** | **0.07** | **0.00** | **0.41** | **0.03** | |
| **Quarantine sheep returning to farm for >3 weeks** | | | | |  |  | |
| Always | 60 | 19.4 | 1.00 |  |  |  | |
| **Sometimes** | **49** | **15.8** | **1.25** | **1.04** | **1.51** | **0.02** | |
| **Never** | **94** | **30.3** | **1.27** | **1.07** | **1.50** | **<0.01** | |
| (Missing) | 107 | 34.5 | 0.94 | 0.78 | 1.14 | 0.53 | |
| **Time to treatment of lambs with SFR** | | | |  |  |  | |
| 0-3 days | 161 | 51.9 | 1.00 |  |  |  | |
| **>3 days** | **131** | **42.3** | **1.27** | **1.07** | **1.50** | **<0.01** | |
| None to treat | 18 | 5.8 | 0.97 | 0.53 | 1.62 | 0.90 | |
| **Stocking density** | | |  |  |  |  | |
| <4 ewes/acre | 164 | 52.9 | 1.00 |  |  |  | |
| **4-8 ewes/acre** | **132** | **42.6** | **1.15** | **1.01** | **1.30** | **0.03** | |
| >8 ewes/acre | 14 | 4.5 | 1.27 | 0.99 | 1.62 | 0.06 | |
| 1. RR = risk ratio, LCI = lower confidence interval, UCI = upper confidence interval, N = number of flocks, SFR = severe footrot 2. Variables highlighted in bold where p<0.05 (Wald’s test of significance) 3. Dispersion parameter taken to be 2.81 | | | | | | |  |

Supplementary Table 4: Multivariable negative binomial generalised linear model for the management practices associated with the proportion of lame ewes in 310 sheep flocks in Great Britain, October 2017-September 2018.

| **Predictor** | **N** | **%** | **RR** | **LCI** | **UCI** | **P-value** | |
| --- | --- | --- | --- | --- | --- | --- | --- |
| (Intercept) |  |  | 0.02 | 0.02 | 0.03 | <0.01 | |
| **Country** |  |  |  |  |  |  | |
| England | 219 | 70.6 | 1.00 |  |  |  | |
| Scotland | 43 | 13.9 | 0.86 | 0.71 | 1.03 | 0.10 | |
| Wales | 48 | 15.5 | 0.83 | 0.69 | 1.01 | 0.06 | |
| **Flock size (number of ewes)** | | | | |  |  | |
| 101-500 | 118 | 38.1 | 1.00 |  |  |  | |
| 1-100 | 91 | 29.4 | 1.20 | 0.96 | 1.50 | 0.11 | |
| 501-1000 | 72 | 23.2 | 0.93 | 0.79 | 1.09 | 0.36 | |
| >1000 | 29 | 9.4 | 0.93 | 0.74 | 1.16 | 0.50 | |
| **Problem flock (Decile 10 -** ≥**7.14% lameness)** | | | |  |  |  | |
| No | 279 | 90.0 | 1.00 |  |  |  | |
| **Yes** | **31** | **10.0** | **3.72** | **2.99** | **4.65** | **<0.01** | |
| **% sheep feet bleeding at routine foot trim** | | | |  |  |  | |
| Did not foot trim | 115 | 37.1 | 1.00 |  |  |  | |
| 0 | 50 | 16.1 | 0.89 | 0.70 | 1.13 | 0.33 | |
| >0-<5 | 104 | 33.5 | 1.02 | 0.87 | 1.19 | 0.85 | |
| **5-100** | **41** | **13.2** | **1.32** | **1.07** | **1.62** | **<0.01** | |
| **Years FootVax™ used** | | |  |  |  |  | |
| Did not vaccinate | 219 | 70.6 | 1.00 |  |  |  | |
| <1 | 10 | 3.2 | 1.33 | 0.97 | 1.85 | 0.08 | |
| 1-<2 | 19 | 6.1 | 1.23 | 0.93 | 1.65 | 0.15 | |
| 2-5 | 32 | 10.3 | 0.86 | 0.69 | 1.07 | 0.16 | |
| **>5** | **30** | **9.7** | **0.72** | **0.57** | **0.90** | **<0.01** | |
| **Footbath to treat SFR** | | | |  |  |  | |
| No | 230 | 74.2 | 1.00 |  |  |  | |
| **Yes** | **80** | **25.8** | **1.17** | **1.01** | **1.36** | **0.04** | |
| **Time to treatment of ewes with SFR** | | | |  |  |  | |
| 0-3 days | 165 | 53.2 | 1.00 |  |  |  | |
| >3 days | 141 | 45.5 | 1.03 | 0.90 | 1.17 | 0.71 | |
| **None to treat** | **4** | **1.3** | **0.08** | **0.01** | **0.29** | **<0.01** | |
| **Predominant soil type - peat** | | | |  |  |  | |
| No | 265 | 85.5 | 1.00 |  |  |  | |
| **Yes** | **45** | **14.5** | **0.79** | **0.65** | **0.95** | **0.01** | |
| **Quarantine new sheep for >3 weeks** | | | | |  |  | |
| Always | 162 | 52.3 | 1.00 |  |  |  | |
| Did not purchase | 34 | 11 | 0.88 | 0.65 | 1.18 | 0.39 | |
| Sometimes | 58 | 18.7 | 1.13 | 0.95 | 1.35 | 0.16 | |
| **Never** | **56** | **18.1** | **1.28** | **1.06** | **1.55** | **0.01** | |
| **Formalin used in footbaths** | | | |  |  |  | |
| Did not footbath | 66 | 21.3 | 1.00 |  |  |  | |
| **Always** | **85** | **27.4** | **1.36** | **1.07** | **1.73** | **0.01** | |
| Sometimes | 79 | 25.5 | 1.20 | 0.94 | 1.54 | 0.13 | |
| Never | 80 | 25.8 | 1.23 | 0.97 | 1.56 | 0.09 | |
| 1. RR = risk ratio, LCI = lower confidence interval, UCI = upper confidence interval, N = number of flocks, SFR = severe footrot 2. Variables highlighted in bold where p<0.05 (Wald’s test of significance) 3. Theta estimated as 5.77 | | | | | | |  |

Supplementary Figure 3: Visual assessment of model fit for the quasi-Poisson (A, B) and negative binomial models (C, D) for associations between management practices and the number of lame ewes in 310 sheep flocks in Great Britain, October 2017-September 2018. Plots A and C show the observed and expected numbers of lame sheep ranked into ten deciles by the observed numbers of lame sheep, while plots B and D show a scatterplot of observed and expected numbers of lame sheep.

Supplementary Table 5: Variables with a stability of >80% and p-value of <0.05 in the bootstrap Poisson elastic net model for the management practices associated with the proportion of lame ewes in 310 sheep flocks in Great Britain, October 2017-September 2018.

| **Predictor** | **N** | **%** | **RR** | **LCI** | **UCI** | **P-value** |
| --- | --- | --- | --- | --- | --- | --- |
| Country: Scotland | 43 | 13.9 | 0.85 | 0.67 | 1.00 | 0.03 |
| Flock size: 1-100 ewes | 91 | 29.4 | 1.12 | 1.00 | 1.34 | 0.02 |
| Footbath to treat ID: yes | 140 | 45.2 | 1.13 | 1.01 | 1.26 | <0.01 |
| Footbath to treat SFR: yes | 80 | 25.8 | 1.13 | 1.00 | 1.38 | 0.01 |
| Formalin used in footbaths: always | 85 | 27.4 | 1.12 | 1.01 | 1.23 | 0.01 |
| Maximum altitude sheep grazed at: >230-500m above sea level | 52 | 16.8 | 0.84 | 0.68 | 0.98 | 0.01 |
| Minimum altitude sheep grazed at: >700-1200m above sea level | 38 | 12.3 | 0.87 | 0.67 | 1.01 | 0.04 |
| Problem flock (Decile 10 - ≥7.14% lameness): yes | 31 | 10.0 | 2.89 | 2.25 | 4.06 | <0.01 |
| Quarantine new sheep to farm for >3 weeks: never | 56 | 18.1 | 1.17 | 1.02 | 1.42 | <0.01 |
| Quarantine sheep returning to farm for >3 weeks: sometimes | 49 | 15.8 | 1.13 | 1.00 | 1.40 | 0.01 |
| Quarantine of sheep returning to farm for >3 weeks: never | 94 | 30.3 | 1.17 | 1.03 | 1.38 | <0.01 |
| Lame sheep separated when gathered: yes | 43 | 13.9 | 1.17 | 1.02 | 1.38 | <0.01 |
| Predominant soil type – peat: yes | 45 | 14.5 | 0.82 | 0.66 | 0.98 | <0.01 |
| Time to treatment of ewes with SFR: none to treat | 4 | 1.3 | 0.43 | 0.15 | 0.83 | <0.01 |
| % sheep feet bleeding at routine trim: 5-100 | 41 | 13.2 | 1.36 | 1.17 | 1.60 | <0.01 |
| Years FootVax™ used: <1 | 10 | 3.2 | 1.42 | 1.09 | 1.84 | <0.01 |
| Years FootVax™ used: >5 | 30 | 9.7 | 0.84 | 0.69 | 0.99 | 0.02 |

1. Variables shown with a bootstrap p-value of <0.05 and stability of >80%.
2. RR = risk ratio, LCI = lower confidence interval, UCI = upper confidence interval, N = number of flocks, SFR = severe footrot, ID = interdigital dermatitis
3. Dummy variables were used, therefore for categorical variables with >2 levels, % indicates the percentage performing the relevant category of the management out of the 310 flocks used for modelling.

Supplementary Table 6: Variables with a stability of >80% and p-value of <0.05 in the bootstrap Gaussian elastic net model for the management practices associated with the proportion of lame ewes in 310 sheep flocks in Great Britain, October 2017-September 2018.

| **Predictor** | **N** | **%** | **Coef** | **LCI** | **UCI** | **P-value** |
| --- | --- | --- | --- | --- | --- | --- |
| Country: Scotland | 43 | 13.9 | -0.07 | -0.16 | 0.00 | <0.01 |
| Foot spray used to treat ewes with SFR: sometimes | 39 | 12.6 | -0.09 | -0.19 | -0.01 | <0.01 |
| Flock size: 1-100 ewes | 91 | 29.4 | 0.24 | 0.16 | 0.33 | <0.01 |
| Flock size: 501-1000 ewes | 72 | 23.2 | -0.05 | -0.14 | 0.00 | 0.04 |
| Flock size: >1000 ewes | 29 | 9.4 | -0.07 | -0.17 | -0.01 | <0.01 |
| Footbath used to treat ID: yes | 140 | 45.2 | 0.08 | 0.01 | 0.13 | <0.01 |
| Formalin used in footbaths: always | 85 | 27.4 | 0.04 | 0.00 | 0.09 | 0.02 |
| Set stocked grazing system used: yes | 147 | 47.4 | -0.03 | -0.11 | 0.00 | 0.04 |
| Maximum altitude sheep grazed at: >230-500m above sea level | 52 | 16.8 | -0.09 | -0.16 | -0.03 | <0.01 |
| Sheep mixed at shows: yes | 23 | 7.4 | 0.08 | 0.00 | 0.18 | 0.02 |
| Open flock: yes | 263 | 84.8 | -0.06 | -0.18 | 0.00 | 0.02 |
| Problem flock (Decile 10 - ≥7.1% lameness): yes | 31 | 10.0 | 0.42 | 0.33 | 0.49 | <0.01 |
| Sheep purchased from market: yes | 200 | 64.5 | -0.05 | -0.12 | 0.00 | <0.01 |
| Sheep purchased from private sale: yes | 110 | 35.5 | -0.05 | -0.13 | 0.00 | 0.01 |
| Quarantine new sheep to farm for >3 weeks: never | 56 | 18.1 | 0.07 | 0.00 | 0.14 | 0.02 |
| Quarantine sheep returning to farm for >3 weeks: never | 94 | 30.3 | 0.05 | 0.01 | 0.12 | 0.02 |
| Source of replacement sheep: purchased + homebred | 94 | 30.3 | 0.04 | 0.00 | 0.09 | 0.02 |
| Selection of replacements from never lame ewes: no | 87 | 28.1 | 0.05 | 0.00 | 0.15 | 0.02 |
| Selection of replacements from never lame ewes: NA (none replaced) | 38 | 12.3 | 0.07 | 0.00 | 0.18 | 0.01 |
| Lame sheep separated when gathered: yes | 43 | 13.9 | 0.11 | 0.02 | 0.22 | <0.01 |
| Predominant soil type - peat: yes | 45 | 14.5 | -0.08 | -0.16 | 0.00 | 0.03 |
| Time to treatment of ewes with SFR: none to treat | 4 | 1.3 | -0.49 | -0.86 | -0.27 | <0.01 |
| % sheep feet bleeding at routine trim: 5-100 | 41 | 13.2 | 0.11 | 0.04 | 0.19 | <0.01 |
| Years FootVax™ used: <1 | 10 | 3.2 | 0.17 | 0.07 | 0.37 | <0.01 |

1. Variables shown with a bootstrap p-value of <0.05 and stability of >80%.
2. Coef = coefficient, LCI = lower confidence interval, UCI = upper confidence interval, N = number of flocks, SFR = severe footrot, ID = interdigital dermatitis
3. Dummy variables were used, therefore for categorical variables with >2 levels, % indicates the percentage performing the relevant category of the management out of the 310 flocks used for modelling.

Supplementary Table 7: Multivariable Quasi-Poisson generalized linear model for the management practices associated with the proportion of lame lambs in 310 sheep flocks in Great Britain, October 2017-September 2018.

| **Predictor** | **N** | **%** | **RR** | **LCI** | **UCI** | **P-value** |  |
| --- | --- | --- | --- | --- | --- | --- | --- |
| (Intercept) |  |  | 0.04 | 0.02 | 0.07 | <0.01 |  |
| **Country** |  |  |  |  |  |  |  |
| England | 219 | 70.6 |  |  |  |  |  |
| **Scotland** | **43** | **13.9** | **0.52** | **0.35** | **0.75** | **<0.01** |  |
| **Wales** | **48** | **15.5** | **0.62** | **0.41** | **0.93** | **0.03** |  |
| **Flock size (number of ewes)** | | |  |  |  |  |  |
| 101-500 | 118 | 38.1 |  |  |  |  |  |
| 1-100 | 91 | 29.4 | 1.04 | 0.54 | 1.85 | 0.91 |  |
| 501-1000 | 72 | 23.2 | 0.98 | 0.70 | 1.37 | 0.90 |  |
| >1000 | 29 | 9.4 | 1.22 | 0.89 | 1.69 | 0.22 |  |
| **Footbath to treat ID** | | | |  |  |  |  |
| No | 170 | 54.8 |  |  |  |  |  |
| **Yes** | **140** | **45.2** | **1.64** | **1.25** | **2.17** | **<0.01** |  |
| **Foot trim to treat lambs with SFR** | | | |  |  |  |  |
| Never | 60 | 19.4 |  |  |  |  |  |
| **Sometimes** | **140** | **45.2** | **0.52** | **0.33** | **0.84** | **<0.01** |  |
| **Always** | **110** | **35.5** | **0.59** | **0.39** | **0.91** | **0.02** |  |
| **Foot trim to treat ewes with SFR** | | | |  |  |  |  |
| Never | 51 | 16.5 |  |  |  |  |  |
| Sometimes | 97 | 31.3 | 1.73 | 0.95 | 3.20 | 0.08 |  |
| **Always** | **162** | **52.3** | **2.13** | **1.24** | **3.68** | **<0.01** |  |
| **Lambs with SFR separated from flock at treatment** | | | | |  |  |  |
| Always | 30 | 9.7 |  |  |  |  |  |
| **Sometimes** | **136** | **43.9** | **0.55** | **0.38** | **0.79** | **<0.01** |  |
| **Never** | **144** | **46.5** | **0.63** | **0.45** | **0.89** | **<0.01** |  |
| **% sheep feet bleeding at routine foot trim** | | | |  |  |  |  |
| Did not foot trim | 115 | 37.1 |  |  |  |  |  |
| 0 | 50 | 16.1 | 0.64 | 0.33 | 1.16 | 0.17 |  |
| >0-<5 | 104 | 33.5 | 1.11 | 0.78 | 1.58 | 0.57 |  |
| **5-100** | **41** | **13.2** | **1.91** | **1.34** | **2.72** | **<0.01** |  |
| **Selection of replacements from ewes that were never lame** | | | | | |  |  |
| Yes | 86 | 27.7 |  |  |  |  |  |
| **No** | **87** | **28.1** | **2.07** | **1.47** | **2.92** | **<0.01** |  |
| **Unknown** | **99** | **31.9** | **1.61** | **1.15** | **2.27** | **<0.01** |  |
| Not applicable | 38 | 12.3 | 1.18 | 0.70 | 1.94 | 0.53 |  |
| **Predominant soil type – peat** | | | |  |  |  |  |
| No | 265 | 85.5 |  |  |  |  |  |
| **Yes** | **45** | **14.5** | **0.53** | **0.35** | **0.78** | **<0.01** |  |
| **Predominant soil type - clay** | | | |  |  |  |  |
| No | 141 | 45.5 |  |  |  |  |  |
| **Yes** | **169** | **54.5** | **1.38** | **1.06** | **1.81** | **0.02** |  |
| **Maximum altitude flock was grazed at (m above sea level)** | | | | |  |  |  |
| 0-230 | 52 | 16.8 |  |  |  |  |  |
| **>230-500** | **52** | **16.8** | **0.49** | **0.31** | **0.78** | **<0.01** |  |
| **>500-850** | **61** | **19.7** | **0.54** | **0.35** | **0.82** | **<0.01** |  |
| >850-1200 | 56 | 18.1 | 0.72 | 0.48 | 1.10 | 0.13 |  |
| **>1200-3400** | **42** | **13.5** | **0.46** | **0.31** | **0.68** | **<0.01** |  |
| (Missing) | 47 | 15.2 | 0.79 | 0.51 | 1.22 | 0.30 |  |
| 1. RR = risk ratio, LCI = lower confidence interval, UCI = upper confidence interval, N = number of flocks, SFR = severe footrot, ID = interdigital dermatitis | | | | | | | |
| 2. Variables highlighted in bold where p<0.05 (Wald's test of significance) | | | | | | | |
| 3. Dispersion parameter taken to be 25.48 | | | | | | | |

Supplementary Table 8: Multivariable negative binomial generalised linear model for the management practices associated with the proportion of lame lambs in 310 sheep flocks in Great Britain, October 2017-September 2018.

| **Predictor** | **N** | **%** | **RR** | **LCI** | **UCI** | **P-value** | |
| --- | --- | --- | --- | --- | --- | --- | --- |
| (Intercept) |  |  | 0.16 | 0.03 | 0.78 | 0.02 | |
| **Country** |  |  |  |  |  |  | |
| England | 219 | 70.6 |  |  |  |  | |
| **Scotland** | **43** | **13.9** | **0.71** | **0.52** | **0.96** | **0.02** | |
| Wales | 48 | 15.5 | 0.84 | 0.64 | 1.12 | 0.23 | |
| **Flock size (number of ewes)** | | | |  |  |  | |
| 101-500 | 91 | 29.4 |  |  |  |  | |
| 1-100 | 118 | 38.1 | 1.34 | 0.99 | 1.82 | 0.05 | |
| 501-1000 | 72 | 23.2 | 0.90 | 0.69 | 1.18 | 0.42 | |
| >1000 | 29 | 9.4 | 1.32 | 0.91 | 1.95 | 0.13 | |
| **Time to treatment of lambs with SFR** | | | |  |  |  | |
| 0-3 days | 161 | 51.9 |  |  |  |  | |
| **>3 days** | **131** | **42.3** | **1.51** | **1.22** | **1.87** | **<0.01** | |
| **None to treat** | **18** | **5.8** | **0.04** | **0.01** | **0.12** | **<0.01** | |
| **Footbath to treat ID** | | | |  |  |  | |
| No | 170 | 54.8 |  |  |  |  | |
| **Yes** | **140** | **45.2** | **1.35** | **1.09** | **1.68** | **<0.01** | |
| **Antibiotic injection to treat lambs with SFR** | | | | | |  | |
| Always | 109 | 35.2 |  |  |  |  | |
| Sometimes | 136 | 43.9 | 0.99 | 0.78 | 1.26 | 0.92 | |
| **Never** | **65** | **21.0** | **0.71** | **0.53** | **0.95** | **0.02** | |
| **Foot trim to treat lambs with SFR** | | | | |  |  | |
| Never | 60 | 19.4 |  |  |  |  | |
| **Sometimes** | **140** | **45.2** | **0.58** | **0.39** | **0.86** | **0.01** | |
| Always | 110 | 35.5 | 0.74 | 0.49 | 1.10 | 0.15 | |
| **Lambs with SFR separated from flock at treatment** | | | | |  |  | |
| Always | 30 | 9.7 |  |  |  |  | |
| **Sometimes** | **136** | **43.9** | **0.64** | **0.44** | **0.93** | **0.01** | |
| **Never** | **144** | **46.5** | **0.71** | **0.49** | **1.00** | **0.04** | |
| **Foot trim to treat ewes with SFR** | | | |  |  |  | |
| Never | 51 | 16.5 |  |  |  |  | |
| **Sometimes** | **97** | **31.3** | **1.70** | **1.09** | **2.63** | **0.03** | |
| **Always** | **162** | **52.3** | **1.95** | **1.26** | **3.01** | **<0.01** | |
| **% sheep feet bleeding at routine foot trim** | | | |  |  |  | |
| Did not foot trim | 115 | 37.1 |  |  |  |  | |
| 0 | 50 | 16.1 | 0.74 | 0.52 | 1.06 | 0.08 | |
| >0-<5 | 104 | 33.5 | 0.90 | 0.69 | 1.18 | 0.43 | |
| **5-100** | **41** | **13.2** | **1.48** | **1.07** | **2.07** | **0.02** | |
| **Selection of replacements from ewes that were never lame** | | | | |  |  | |
| Yes | 86 | 27.7 |  |  |  |  | |
| **No** | **87** | **28.1** | **1.77** | **1.34** | **2.34** | **<0.01** | |
| **Unknown** | **99** | **31.9** | **1.38** | **1.04** | **1.84** | **0.02** | |
| Not applicable | 38 | 12.3 | 1.06 | 0.64 | 1.74 | 0.81 | |
| **Source of replacement sheep** | | | | |  |  | |
| Homebred | 164 | 52.9 |  |  |  |  | |
| Purchased | 42 | 13.5 | 1.40 | 0.95 | 2.09 | 0.09 | |
| **Homebred + purchased** | **94** | **30.3** | **1.55** | **1.21** | **1.97** | **<0.01** | |
| Not applicable | 10 | 3.2 | 1.84 | 0.93 | 3.80 | 0.09 | |
| **Predominant soil type - peat** | | | |  |  |  | |
| No | 265 | 85.5 |  |  |  |  | |
| **Yes** | **45** | **14.5** | **0.64** | **0.48** | **0.87** | **<0.01** | |
| **Maximum altitude flock was grazed at (m above sea level)** | | | | |  |  | |
| 0-230 | 52 | 16.8 |  |  |  |  | |
| **>230-500** | **52** | **16.8** | **0.69** | **0.48** | **0.99** | **0.04** | |
| >500-850 | 61 | 19.7 | 0.74 | 0.52 | 1.05 | 0.08 | |
| >850-1200 | 56 | 18.1 | 0.88 | 0.60 | 1.28 | 0.48 | |
| **>1200-3400** | **42** | **13.5** | **0.63** | **0.42** | **0.95** | **0.03** | |
| (Missing) | 47 | 15.2 | 1.12 | 0.78 | 1.63 | 0.53 | |
| **Pasture used as forage** | | | |  |  |  | |
| No | 2 | 0.6 |  |  |  |  | |
| **Yes** | **308** | **99.4** | **0.16** | **0.03** | **0.74** | **0.01** | |
| **Flock mixed with others at shows** | | | |  |  |  | |
| No | 287 | 92.6 |  |  |  |  | |
| **Yes** | **23** | **7.4** | **0.63** | **0.43** | **0.96** | **0.03** | |
| 1. RR = risk ratio, LCI = lower confidence interval, UCI = upper confidence interval, N = number of flocks, SFR- severe footrot, ID = interdigital dermatitis | | | | | | |  |
| 2. Variables highlighted in bold where p<0.05 (Wald's test of significance) | | | | | | |  |
| 3. Theta taken to be 1.89 | | | | | | |  |

Supplementary Figure 4: Visual assessment of model fit for the quasi-Poisson (A, B) and negative binomial models (C, D) for associations between management practices and the number of lame lambs in 310 sheep flocks in Great Britain, October 2017-September 2018. Plots A and C show the observed and expected numbers of lame sheep ranked into ten deciles by the observed numbers of lame sheep, while plots B and D show a scatterplot of observed and expected numbers of lame sheep.

Supplementary Table 9: Variables with a stability of >80% and p-value of <0.05 in the bootstrap Poisson elastic net model for the management practices associated with the proportion of lame ewes in 310 sheep flocks in Great Britain, October 2017-September 2018.

| **Predictor** | **N** | **%** | **RR** | **LCI** | **UCI** | **P-value** |
| --- | --- | --- | --- | --- | --- | --- |
| Country: Scotland | 43 | 13.9 | 0.84 | 0.66 | 0.97 | <0.01 |
| Antibiotic injection to treat ewes with SFR: never | 48 | 15.5 | 0.87 | 0.79 | 1.00 | 0.02 |
| Foot spray to treat ewes with SFR: never | 10 | 3.2 | 0.79 | 0.39 | 0.98 | 0.01 |
| Foot trim to treat ewes with SFR: always | 162 | 31.3 | 1.12 | 0.98 | 1.25 | 0.03 |
| Flock size: 501-1000 ewes | 72 | 23.2 | 0.90 | 0.77 | 1.01 | 0.04 |
| Footbath to treat ID: yes | 140 | 45.2 | 1.22 | 1.07 | 1.57 | <0.01 |
| Formalin used in footbaths: always | 85 | 27.4 | 1.14 | 1.02 | 1.31 | <0.01 |
| Flock moved to fresh pasture: other frequency | 153 | 49.4 | 0.89 | 0.76 | 1.00 | 0.02 |
| Antibiotic injection to treat lambs with SFR: never | 65 | 21.0 | 0.92 | 0.71 | 1.00 | 0.04 |
| Maximum altitude sheep grazed at: >230-500ft above sea level | 52 | 16.8 | 0.86 | 0.59 | 0.98 | 0.02 |
| Sheep did not mix with other flocks: yes | 247 | 79.7 | 1.14 | 1.01 | 1.51 | 0.01 |
| Problem flock (Decile 10 - ≥8.5% lameness): yes | 31 | 10.0 | 3.63 | 2.61 | 5.53 | <0.01 |
| Sheep purchased from other source: yes | 13 | 4.2 | 0.80 | 0.50 | 0.97 | <0.01 |
| Quarantine sheep returning to farm for >3 weeks: missing | 107 | 34.5 | 0.91 | 0.78 | 1.02 | 0.04 |
| Source of replacement sheep: purchased + homebred | 94 | 30.3 | 1.12 | 0.98 | 1.26 | 0.05 |
| Selection of replacements from never lame ewes: no | 87 | 28.1 | 1.25 | 1.06 | 1.60 | <0.01 |
| Sheep separated from flock when persistently lame: yes | 119 | 38.4 | 1.14 | 1.01 | 1.27 | 0.02 |
| Predominant soil type – peat: yes | 45 | 14.5 | 0.84 | 0.68 | 0.98 | 0.01 |
| Time to treatment of ewes with SFR: >3 days | 141 | 45.5 | 1.19 | 1.02 | 1.36 | <0.01 |
| Time to treatment of ewes with SFR: none to treat | 4 | 1.3 | 0.64 | 0.28 | 0.96 | <0.01 |
| Time to treatment of lambs with SFR: >3 days | 131 | 42.3 | 1.15 | 1.02 | 1.35 | 0.01 |
| Time to treatment of lambs with SFR: none to treat | 18 | 5.8 | 0.66 | 0.12 | 0.95 | <0.01 |
| % sheep feet bleeding at routine trim: 5-100 | 41 | 13.2 | 1.19 | 1.01 | 1.48 | 0.01 |

1. Variables shown with a bootstrap p-value of <0.05 and stability of >80%.
2. RR = risk ratio, LCI = lower confidence interval, UCI = upper confidence interval, N = number of flocks, SFR = severe footrot, ID = interdigital dermatitis
3. Dummy variables were used, therefore for categorical variables with >2 levels, % indicates the percentage performing the relevant category of the management out of the 310 flocks used for modelling.

Supplementary Table 10: Variables with a stability of >80% and p-value of <0.05 in the bootstrap Gaussian elastic net model for the management practices associated with the proportion of lame ewes in 310 sheep flocks in Great Britain, October 2017-September 2018.

| **Predictor** | **N** | **%** | **Coef** | **LCI** | **UCI** | **P-value** |
| --- | --- | --- | --- | --- | --- | --- |
| Country: Scotland | 43 | 13.9 | -0.07 | -0.19 | -0.01 | 0.01 |
| Culling management: after lame three times/year | 28 | 9.0 | 0.06 | 0.00 | 0.18 | 0.03 |
| Flock size: 1-100 ewes | 118 | 38.1 | 0.17 | 0.09 | 0.30 | <0.01 |
| Flock size: 501-1000 ewes | 72 | 23.2 | -0.08 | -0.14 | -0.01 | 0.01 |
| Footbath to treat ID: yes | 140 | 45.2 | 0.09 | 0.03 | 0.18 | <0.01 |
| Pasture used as forage: yes | 308 | 99.4 | -0.39 | -0.60 | -0.06 | <0.01 |
| Formalin used in footbaths: always | 85 | 27.4 | 0.05 | 0.00 | 0.12 | 0.02 |
| Antibiotic injection to treat lambs with SFR: never | 65 | 21.0 | -0.05 | -0.15 | 0.00 | 0.01 |
| Maximum altitude sheep grazed at: >230-500ft above sea level | 52 | 16.8 | -0.07 | -0.21 | 0.00 | <0.01 |
| Maximum altitude sheep grazed at: >850-1200ft above sea level | 56 | 18.1 | 0.06 | 0.00 | 0.16 | 0.02 |
| Problem flock (Decile 10 - ≥8.5% lameness): yes | 31 | 10.0 | 0.60 | 0.46 | 0.75 | <0.01 |
| Sheep purchased from private sale: yes | 110 | 35.5 | -0.07 | -0.14 | 0.00 | <0.01 |
| Quarantine new sheep to farm for >3 weeks: never | 56 | 18.1 | 0.09 | 0.02 | 0.18 | 0.01 |
| Source of replacement sheep: purchased + homebred | 94 | 30.3 | 0.07 | 0.01 | 0.13 | <0.01 |
| Selection of replacements from never lame ewes: no | 87 | 28.1 | 0.08 | 0.01 | 00.22 | 0.01 |
| Selection of replacements from never lame ewes: unknown | 99 | 31.9 | 0.05 | 0.00 | 0.15 | 0.04 |
| Selection of replacements from never lame ewes: NA (none replaced) | 38 | 12.3 | 0.11 | 0.02 | 0.26 | <0.01 |
| Lame sheep separated when gathered: yes | 43 | 13.9 | 0.07 | 0.00 | 0.20 | 0.02 |
| Predominant soil type – peat: yes | 45 | 14.5 | -0.08 | -0.19 | -0.01 | 0.02 |
| Time to treatment of ewes with SFR: >3 days | 141 | 45.5 | 0.06 | 0.01 | 0.13 | <0.01 |
| Time to treatment of ewes with SFR: none to treat | 4 | 1.3 | -0.45 | -1.03 | -0.07 | <0.01 |
| Time to treatment of lambs with SFR: >3 days | 131 | 42.3 | 0.06 | 0.01 | 0.15 | <0.01 |
| Time to treatment of lambs with SFR: none to treat | 18 | 5.8 | -0.37 | -0.61 | -0.15 | <0.01 |
| Years FootVax™ used: 1-<2 | 19 | 6.1 | 0.12 | 0.00 | 0.28 | 0.02 |
| Years FootVax™ used: 2-5 | 32 | 10.3 | -0.07 | -0.17 | 0.00 | 0.01 |

1. Variables shown with a bootstrap p-value of <0.05 and stability of >80%.
2. Coef = coefficient, LCI = lower confidence interval, UCI = upper confidence interval, N = number of flocks, SFR = severe footrot, ID = interdigital dermatitis
3. Dummy variables were used, therefore for categorical variables with >2 levels, % indicates the percentage performing the relevant category of the management out of the 310 flocks used for modelling.

Supplementary Table 11: the percentages of farmers using Formalin in footbaths for each type of foot lesion (interdigital dermatitis, severe footrot, contagious ovine digital dermatitis, granuloma, shelly hoof and white line abscess) in 310 flocks of sheep in Great Britain (October 2017-September 2018)

| **Lesion** | **Presence** | **N** | **Use of Formalin in footbaths** | | | | |
| --- | --- | --- | --- | --- | --- | --- | --- |
|  |  |  | **Did not footbath** | **Always** | **Sometimes** | **Never** |  |
| Interdigital dermatitis* | Present | 281 | 17.8 | 27.8 | 28.1 | 26.3 |  |
|  | Absent | 22 | 63.6 | 18.2 | 0.0 | 18.2 |  |
|  | Unknown | 0 | 0.0 | 0.0 | 0.0 | 0.0 |  |
|  | Missing | 7 | 28.6 | 42.9 | 0.0 | 28.6 |  |
| Severe footrot* | Present | 242 | 16.9 | 30.2 | 27.3 | 25.6 |  |
|  | Absent | 64 | 37.5 | 15.6 | 20.3 | 26.6 |  |
|  | Unknown | 1 | 0.0 | 0.0 | 0.0 | 100.0 |  |
|  | Missing | 3 | 33.3 | 66.7 | 0.0 | 0.0 |  |
| Contagious ovine digital dermatitis* | Present | 124 | 8.1 | 29.0 | 36.3 | 26.6 |  |
|  | Absent | 172 | 29.7 | 27.3 | 17.4 | 25.6 |  |
|  | Unknown | 9 | 22.2 | 11.1 | 44.4 | 22.2 |  |
|  | Missing | 5 | 60.0 | 20.0 | 0.0 | 20.0 |  |
| Granuloma* | Present | 152 | 13.8 | 31.6 | 28.3 | 26.3 |  |
|  | Absent | 147 | 28.6 | 23.1 | 24.5 | 23.8 |  |
|  | Unknown | 3 | 0.0 | 33.3 | 0.0 | 66.7 |  |
|  | Missing | 8 | 37.5 | 25.0 | 0.0 | 37.5 |  |
| Shelly Hoof | Present | 191 | 19.7 | 32.1 | 24.4 | 23.8 |  |
|  | Absent | 104 | 24.0 | 19.2 | 26.9 | 29.8 |  |
|  | Unknown | 7 | 28.6 | 0.0 | 57.1 | 14.3 |  |
|  | Missing | 6 | 16.7 | 50.0 | 0.0 | 33.3 |  |
| White Line Abscess | Present | 97 | 15.5 | 26.8 | 33.0 | 24.7 |  |
|  | Absent | 188 | 22.9 | 28.2 | 22.3 | 26.6 |  |
|  | Unknown | 15 | 26.7 | 20.0 | 26.7 | 26.7 |  |
|  | Missing | 10 | 40.0 | 30.0 | 10.0 | 20.0 |  |

1. * indicates p <0.05 from a Fisher’s exact test of association between the presence of a foot lesion (presence, absence, unknown, missing) and use of Formalin in footbaths (always, sometimes and never and did not footbath at all). P values obtained by 2000 Monte Carlo simulations.

Supplementary Table 12: Relationships between the percentage of farmers using a treatment type (foot spray, foot trimming, and antibiotic injection) and the length of time taken to treat sheep following recognition of lameness in in 310 flocks of sheep in Great Britain (October 2017-September 2018)

| **Treatment type** | **Frequency of use** | **N** | **Time to treatment** | | |
| --- | --- | --- | --- | --- | --- |
|  |  |  | **0-3** | **>3** | **None to treat** |
|  |  | *Ewes* |  |  |  |
| Antibiotic injection to treat ewes with SFR | Always | 164 | 50.6 | 49.4 | 0.0 |
|  | Sometimes | 98 | 59.2 | 38.8 | 2.0 |
|  | Never | 48 | 50.0 | 45.8 | 4.2 |
| Foot trim to treat ewes with SFR | Always | 162 | 50.6 | 49.4 | 0.0 |
|  | Sometimes | 97 | 55.7 | 41.2 | 3.1 |
|  | Never | 51 | 56.9 | 41.2 | 2.0 |
| Foot spray to treat ewes with SFR | Always | 261 | 53.3 | 46.4 | 0.4 |
|  | Sometimes | 39 | 59.0 | 35.9 | 5.1 |
|  | Never | 10 | 30.0 | 60.0 | 10.0 |
|  |  | *Lambs* |  |  |  |
| Antibiotic injection to treat lambs with SFR | Always | 109 | 52.3 | 47.7 | 0.0 |
|  | Sometimes | 136 | 52.2 | 36.8 | 11.0 |
|  | Never | 65 | 50.8 | 44.6 | 4.6 |
| Foot trim to treat lambs with SFR | Always | 110 | 52.7 | 47.3 | 0.0 |
|  | Sometimes | 140 | 45.7 | 42.9 | 11.4 |
|  | Never | 60 | 65.0 | 31.7 | 3.3 |
| Foot spray to treat lambs with SFR | Always | 239 | 53.1 | 46.9 | 0.0 |
|  | Sometimes | 60 | 46.7 | 26.7 | 26.7 |
|  | Never | 11 | 54.5 | 27.3 | 18.2 |

1. N: number of flocks, SFR: severe footrot

Supplementary Table 13: associations between type of treatment used to treat ewes and lambs with severe footrot in 310 flocks of sheep in Great Britain, October 2017-September 2018)

| **Treatment of ewes and lambs with severe footrot** | **1** | **2** | **3** | **4** | **5** | **6** | **7** | **8** |
| --- | --- | --- | --- | --- | --- | --- | --- | --- |
| **1. Time to treatment of ewes** | - | 0.05 | 0.13 | 0.01 | <0.01 | 0.20 | 0.41 | 0.03 |
| **2. Antibiotic injection to treat ewes with SFR** |  | - | <0.01 | <0.01 | <0.01 | <0.01 | 0.01 | <0.01 |
| **3. Foot trim to treat ewes with SFR** |  |  | - | <0.01 | 0.37 | <0.01 | <0.01 | <0.01 |
| **4. Foot spray to treat ewes with SFR** |  |  |  | - | 0.22 | <0.01 | <0.01 | <0.01 |
| **5. Time to treatment of lambs** |  |  |  |  | - | <0.01 | <0.01 | <0.01 |
| **6. Antibiotic injection to treat lambs with SFR** |  |  |  |  |  | - | <0.01 | <0.01 |
| **7. Foot trim to treat lambs with SFR** |  |  |  |  |  |  | - | <0.01 |
| **8. Foot spray to treat lambs with SFR** |  |  |  |  |  |  |  | - |

1. P-values from chi-square test of association, or Fisher’s exact test (2000 Monte Carlo simulations) where assumptions for the chi square test were not met
2. Categories for type of treatment were always, sometimes and never.
3. Categories for time to treatment were 0-3 days, >3 days and no lame sheep to treat.

Supplementary Table 14a: The number and percentage of farms with <2, 2-5% and >5% prevalence of lameness in both ewes and lambs in 310 flocks of sheep in Great Britain, October 2017-September 2018)

| **Prevalence of lameness** | **Overall** | | **Ewes** | | **Lambs** | |
| --- | --- | --- | --- | --- | --- | --- |
|  | **N** | **%** | **N** | **%** | **N** | **%** |
| <2% | 120 | 38.7 | 109 | 35.2 | 129 | 41.6 |
| 2-5% | 118 | 38.1 | 126 | 40.6 | 110 | 35.5 |
| >5% | 72 | 23.1 | 75 | 24.2 | 71 | 22.9 |

1. N = number, % = percentage

Supplementary Table 14b: The number and percentage of farms with <2, 2-5% and >5% prevalence of lameness in both ewes and lambs in all 450 flocks of sheep in Great Britain, October 2017-September 2018)

| **Prevalence of lameness** | **Overall** | | **Ewes** | | **Lambs** | |
| --- | --- | --- | --- | --- | --- | --- |
|  | **N** | **%** | **N** | **%** | **N** | **%** |
| <2% | 183 | 40.7 | 172 | 38.2 | 195 | 43.3 |
| 2-5% | 169 | 37.6 | 178 | 39.6 | 150 | 33.3 |
| >5% | 98 | 21.8 | 100 | 22.2 | 105 | 23.3 |

1. N = number, % = percentage

Supplementary Table 15: sample sizes required for detection of a range of differences between two groups for the log10(1+x) outcome variable, with a power of 0.80 and alpha of 0.05.

| **Difference between groups to detect** | **Tested standard deviation** | **Sample size required** |
| --- | --- | --- |
| 0.06 | 0.20 | 350 |
| 0.07 | 0.20 | 258 |
| 0.08 | 0.20 | 198 |
| 0.09 | 0.20 | 156 |
| 0.10 | 0.20 | 128 |
| 0.15 | 0.20 | 58 |
| 0.09 | 0.30 | 350 |
| 0.10 | 0.30 | 234 |
| 0.15 | 0.30 | 128 |
| 0.09 | 0.35 | 476 |
| 0.10 | 0.35 | 386 |
| 0.15 | 0.35 | 172 |
| 0.09 | 0.40 | 622 |
| 0.10 | 0.40 | 504 |
| 0.15 | 0.40 | 226 |

1. Difference between groups and standard deviations are on the log10(x+1) scale
